# **scNODE** : generative model for temporal single cell transcriptomic data prediction

**DOI:** 10.1093/bioinformatics/btae393

**Published:** 2024-09-04

**Authors:** Jiaqi Zhang, Erica Larschan, Jeremy Bigness, Ritambhara Singh

**Affiliations:** Department of Computer Science, Brown University, Providence, RI 02906, United States; Center for Computational Molecular Biology, Brown University, Providence, RI 02912, United States; Department of Molecular Biology, Cell Biology and Biochemistry, Brown University, Providence, RI 02912, United States; Center for Computational Molecular Biology, Brown University, Providence, RI 02912, United States; Department of Computer Science, Brown University, Providence, RI 02906, United States; Center for Computational Molecular Biology, Brown University, Providence, RI 02912, United States

## Abstract

**Summary:**

Measurement of single-cell gene expression at different timepoints enables the study of cell development. However, due to the resource constraints and technical challenges associated with the single-cell experiments, researchers can only profile gene expression at discrete and sparsely sampled timepoints. This missing timepoint information impedes downstream cell developmental analyses. We propose scNODE, an end-to-end deep learning model that can predict *in silico* single-cell gene expression at unobserved timepoints. scNODE integrates a variational autoencoder with neural ordinary differential equations to predict gene expression using a continuous and nonlinear latent space. Importantly, we incorporate a dynamic regularization term to learn a latent space that is robust against distribution shifts when predicting single-cell gene expression at unobserved timepoints. Our evaluations on three real-world scRNA-seq datasets show that scNODE achieves higher predictive performance than state-of-the-art methods. We further demonstrate that scNODE’s predictions help cell trajectory inference under the missing timepoint paradigm and the learned latent space is useful for *in silico* perturbation analysis of relevant genes along a developmental cell path.

**Availability and implementation:**

The data and code are publicly available at https://github.com/rsinghlab/scNODE.

## 1 Introduction

A fundamental challenge in biology is understanding how gene expression changes over time during cell development (Moris *et al.* 2016, Fleck *et al.* 2022). With the advent of high-throughput single-cell RNA sequencing (scRNA-seq), researchers now routinely profile gene expression at single-cell resolution (Hwang *et al.* 2018, Chen *et al.* 2019), revealing substantial heterogeneity within the same tissue type or developmental stage (Chen *et al.* 2022a, Ding *et al.* 2022). Profiling scRNA-seq data at multiple timepoints allows us to understand how biological processes unfold within cell populations by inferring cellular trajectories, identifying cell fate transitions, and characterizing differentially expressed genes (Liu *et al.* 2022, Qiu *et al.* 2022).

**Figure 1.**
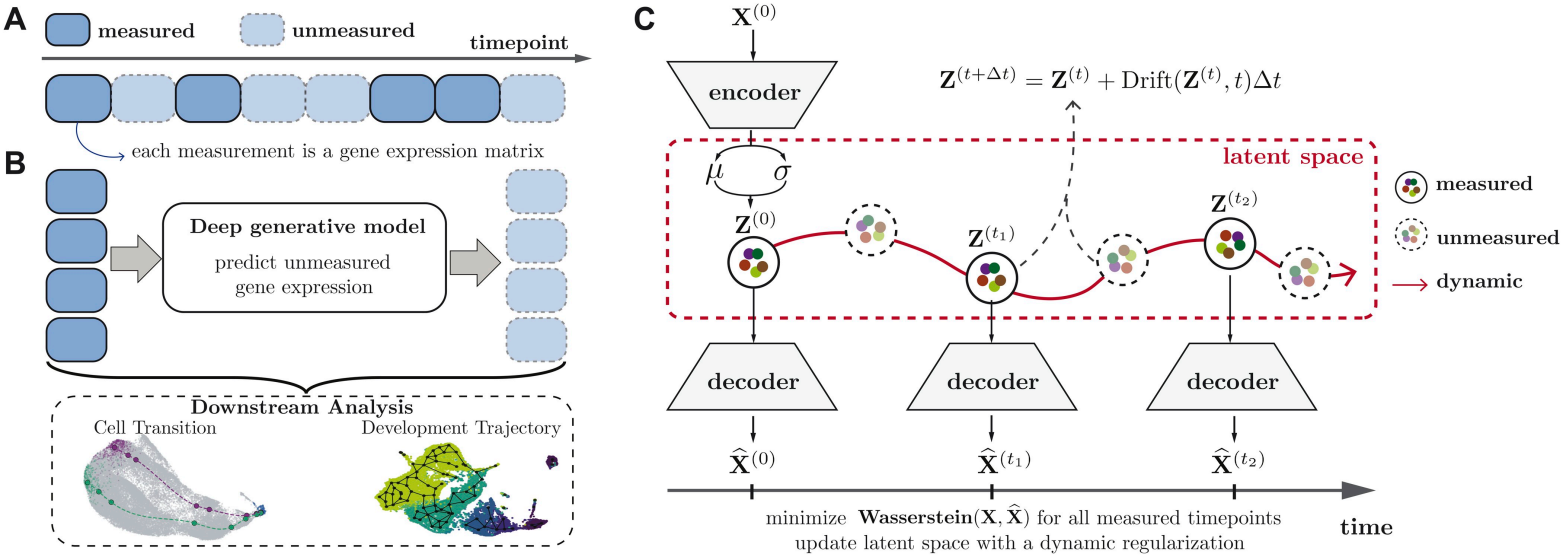
Model overview. (A) The measurements in temporally recorded single-cell data are sparse and unaligned, such that some timepoints are not measured and measurements at different timepoints contain different sets of cells. The datasets in this study sample cellular populations at uniformly spaced time points, although uniform spacing is not a requirement (as depicted in this graphic). (B) scNODE is used to predict expression at unmeasured timepoints. Predictions can be used to analyze cell state transitions and developmental trajectories. (C) scNODE uses VAEs to find an optimal low-dimensional representation. scNODE then uses this representation to solve neural ODEs and predict gene expression at any timepoint $t=t_{1},t_{2},\ldots$ starting from the first timepoint *t *=* *0. Optimal models are fitted by minimizing the Wasserstein distance between observations and predictions at measured timepoints. scNODE also introduces a dynamic regularization to learn a latent space robust to distribution shift.

Temporal scRNA-seq experiments, however, have significant limitations that impede developmental studies. Due to the substantial time and resources involved in conducting these experiments, researchers generally only profile gene expression at discrete and sparsely sampled timepoints (Ding *et al.* 2022). Since it is infeasible to make continuous-time observations, this results in information loss between consecutively measured discrete timepoints. Therefore, it is crucial to develop a model that predicts realistic *in silico* gene expression at any timepoint, whether between (interpolation) or beyond the measured time intervals (extrapolation), to enable single-cell temporal analyses (Fig. 1A and B).

Single-cell gene expression prediction has been investigated in previous studies (Cao *et al.* 2021) through various generative models. Existing methods use generative adversarial networks (GANs) (Marouf *et al.* 2020) or statistical frameworks (Zappia *et al.* 2017, Risso *et al.* 2018, Li and Li 2019, Sun *et al.* 2021) to fit characteristics of the observed single-cell gene expression and augment the dataset. However, these methods are designed for single-cell gene expression measured at a single time snapshot and do not consider developmental dynamics.

Several computational methods have been developed to learn gene expression dynamics by inferring cell trajectories (Trapnell *et al.* 2014, Qiu *et al.* 2017, Herman *et al.* 2018, Saelens *et al.* 2019, Wolf *et al.* 2019, Li 2023) from temporal scRNA-seq. However, most of them infer pseudo-times, which analyze cell differentiation based on geometric distances in low-dimensional spaces. Hence, they do not accurately model how cells develop in real physical time. Sagittarius method (Woicik *et al.* 2023) predicts gene expressions at future timepoints but requires cell matching between timepoints. This matching is hard to obtain for real-world scRNA-seq as the cells are lysed during the experiment. RNA velocity based methods (La Manno *et al.* 2018, Bergen *et al.* 2021, 2020) are used to uncover developmental trends and underlying kinetics of gene expression. These methods require deeply sequenced datasets to obtain spliced and unspliced counts as prior information, and most of them are nongenerative methods. Waddington-OT (Schiebinger *et al.* 2019) and TrajectoryNet (Tong *et al.* 2020) interpolate single-cell gene expression between two timepoints in a developmental trajectory but are unable to extrapolate beyond the observed timepoints.

Recently, a few generative methods have modeled cell differentiation trajectory and predicted gene expression at multiple unobserved time points. For example, PRESCIENT (Yeo *et al.* 2021) applies principal component analysis (PCA) to reduce high-dimensional gene space to a low-dimensional representation. It then applies neural ordinary differential equations (ODEs) (Chen *et al.* 2018) in this low-dimensional space to model the cell developmental trajectory. Multiple studies (Ding *et al.* 2018, Tran *et al.* 2021, Xiang *et al.* 2021) have found that PCA-based low-dimensional representations of scRNA-seq expression cannot capture complex relationships in the highly heterogeneous single-cell data. Thus, PCA may have the issue of overcrowding representation (Kobak and Berens 2019, Tran *et al.* 2021), where cells of different types are poorly separated in PCA-based low-dimensional space, and cellular variations are lost. MIOFlow (Huguet *et al.* 2022) replaces PCA with a geodesic variational autoencoder (VAE) to learn nonlinear low-dimensional representations that better retain cellular variations. However, this method fixes its low-dimensional space at the beginning, followed by neural ODE modeling. We hypothesize that a fixed low-dimensional representation obtained using observed timepoints may not generalize to an unobserved timepoint, if its underlying distribution differs substantially.

We propose a single-cell neural ODE (scNODE) framework, which integrates a VAE (Kingma and Welling 2019) with neural ODE to predict accurate gene expression at any unmeasured timepoint using a dynamic low-dimensional space (Fig. 1C). First, scNODE uses VAE to obtain a low-dimensional representation that retains cellular variations. scNODE then predicts single-cell gene expression for unmeasured timepoints with a neural ODE. Importantly, scNODE uses a *dynamic regularization* term to incorporate the dynamic manifold learned from neural ODE as a prior. Therefore, the learned VAE space is robust to the distribution shift between observed and unobserved time points. We evaluate scNODE on three real-world scRNA-seq datasets. Our results demonstrate that scNODE predicts single-cell gene expression more accurately than state-of-the-art methods for unobserved timepoints (interpolation and extrapolation). We further show that scNODE gene expression predictions assist with cell developmental trajectory inference. Finally, scNODE learns an interpretable low-dimensional space, which enables conducting *in silico* perturbation analysis of relevant genes to study cell development.

## 2 Materials and methods

*Notation:* Let $X^{\left(t\right)}\in R^{n_{t}\times p}$ denote the gene expression matrix at the *t*th time point of *n_t_* cells by *p* genes. Given gene expression measurements at observed timepoints ${\left\{X^{\left(t\right)}\right\}}_{t\in T}$, our goal is to predict gene expression at any timepoint. Here, T⊆{0,1,2,…} denotes the observed timepoint indices. Note that we assume the same set of highly variable genes (HVGs) is measured at each time point, but due to the destruction of cells during scRNA experiments, a different set of cells is measured at each time point. Furthermore, let $Z^{\left(t\right)}\in R^{n_{t}\times d}$ denote the *d*-dimensional (d≪p) latent variable at the *t*th time point.

### 2.1 scNODE uses VAE to learn complex low-dimensional space

scNODE uses a VAE to learn a low-dimensional representation (also known as latent space) of the single-cell gene expression measurements at the observed timepoints. A VAE is a neural network-based generative model that maps high-dimensional data to a low-dimensional representation and is widely used in many single-cell studies (Wang and Gu 2018, Grønbech *et al.* 2020, Lin *et al.* 2020, Svensson *et al.* 2020), showing superior performance. The benefit of using VAEs for single-cell data over PCA is that the nonlinearity of neural networks can more effectively capture complex cell relationships and cellular variations.

scNODE first pre-trains a VAE to obtain a latent space that captures the gene expression of single cells at all available training timepoints. Specifically, scNODE trains the VAE component to perform multi-timepoint modeling by inputting the gene expression of all observed cells $X_{\text{ALL}}=\text{CONCAT}\left(X^{\left(t\right)}\;|\;t\in T\right)$. VAE is composed of two neural networks: (i) the encoder network ${\text{Enc}}_{\phi }$ maps expression from gene space $R^{p}$ to a low-dimensional latent space $R^{d}$, parameterized by a Gaussian distribution N(μ,σ), and (ii) the decoder network ${\text{Dec}}_{\theta }$ maps samples in the latent space to gene space in the opposite direction to reconstruct the input. Given $X_{\text{ALL}}$, VAE learns latent representations through

(1) $$\begin{array}{c} \mu_{\text{ALL}},\sigma_{\text{ALL}}={\text{Enc}}_{\phi }(X_{\text{ALL}}),\\ Z_{\text{ALL}}∼N(\mu_{\text{ALL}},\sigma_{\text{ALL}}),\\ {\hat{X}}_{\text{ALL}}={\text{Dec}}_{\theta }(Z_{\text{ALL}}). \end{array}$$

The encoder and decoder networks are parameterized by ϕ and *θ*, correspondingly. VAE minimizes a combination of the (i) mean squared error (MSE) between input gene expression and the reconstructed gene expression from the decoder and (ii) the Kulback-Leibler (KL) divergence (Csiszár 1975) between the latent distribution and a standard Gaussian prior:

(2) $$\begin{array}{c} L_{\text{pre}}=\text{MSE}\left(X_{\text{ALL}},\;{\hat{X}}_{\text{ALL}}\right)+\lambda \text{KL}\left(N(\mu_{\text{ALL}},\sigma_{\text{ALL}}),\;N(0,1)\right). \end{array}$$

KL divergence is an information-theoretic quantity that quantifies the distance between two probability distributions. By using a Gaussian prior, the KL divergence forces the encoder to find latent representations of the cells that are well-separated. scNODE pre-trains the VAE component with Equation (2), such that the encoder latent space preserves the variation of all observed cells. Next, we model the developmental dynamics of this latent space.

### 2.2 scNODE uses neural ODE to model cell dynamics

scNODE uses ODEs to model cell developmental dynamics in the latent space learned by the VAE. An ODE is an equation describing how a quantity *x* changes with respect to an independent variable *y*, such that dx=f(x;y)dy where function *f* represents the derivative. Therefore, we can use differential equations to model how gene expression changes with respect to time. However, for the high-dimensional data, finding the solution of the derivative function *f* through numerical methods is intractable and computationally expensive (Kidger 2021). Therefore, recent studies adopt neural networks to approximate the derivative function and have proposed neural ODEs (Chen *et al.* 2018). Neural ODEs (formulated with respect to time) are useful for constructing continuous-time trajectories and have been explored before to model single-cell development (Matsumoto *et al.* 2017, Chen *et al.* 2022b, Qiu *et al.* 2022).

scNODE uses neural ODEs to parameterize the continuous dynamics of gene expression in the latent space. Specifically, scNODE quantifies changes of cell latent representation $Z^{\left(t\right)}$ (from VAE encoding) at time *t* through a neural ODE

(3) $$dZ^{\left(t\right)}={\text{Drift}}_{\omega }\left(Z^{\left(t\right)};t\right)\cdot dt$$

Here, ${\text{Drift}}_{\omega }$ is a nonlinear neural network with parameterization *ω*, modeling the developmental velocities in the latent space, such that ${\text{Drift}}_{\omega }(Z^{\left(t\right)};t)$ represents the direction and strength of cellular transitions. scNODE computes the latent representation $Z^{\left(0\right)}$ of cells at the first timepoint *t *=* *0 through the encoder network (pre-trained in the previous step) and predicts the subsequent cell states step-wise at any timepoint *t* as follows:

(4) $$\begin{array}{c} Z^{\left(0\right)}∼N(\mu_{0},\sigma_{0})\;\text{and}\;\mu_{0},\sigma_{0}={\text{Enc}}_{\phi }(X^{\left(0\right)}),\\ Z^{\left(t+\Delta t\right)}=Z^{\left(t\right)}+{\text{Drift}}_{\omega }\left(Z^{\left(t\right)},t\right)\Delta t. \end{array}$$

Here, hyperparameter Δt denotes step size and drift term ${\text{Drift}}_{\omega }\left(Z^{\left(t\right)},t\right)\Delta t$ represents the forward steps taken in the latent space. While there are several methods that can solve this ODE, in this work, we apply the commonly used first-order Euler method [in Equation (4)] for convenience of explanation. However, in our implementation, one can specify any ODE solver.

To fit the continuous trajectory (controlled by ${\text{Drift}}_{\omega }$) to the observations, scNODE minimizes the difference between the input and the reconstructed gene expression. Specifically, at each measured timepoint t∈T, scNODE uses the decoder ${\text{Dec}}_{\theta }$ to convert latent variables $Z^{\left(t\right)}$ generated from Equation (4) back to the gene space through ${\hat{X}}^{\left(t\right)}={\text{Dec}}_{\theta }\left(Z^{\left(t\right)}\right)$. Because we have no correspondence between true cells and cells generated from the ODE model, scNODE utilizes the Wasserstein metric (Cuturi 2013) to measure the distance between distributions defined by ground truth **X** and predictions $\hat{X}$ as

(5) $$\begin{array}{c} \text{Wass}(X,\hat{X})={\left(\underset{\Gamma ∼\Pi (X,\hat{X})}{\min}\sum_{i,j}D_{ij}^{2}\Gamma_{ij}\right)}^{1/2}\;\text{with}\\ D_{ij}=||X_{i}-{\hat{X}}_{j}|{|}_{2}. \end{array}$$

Here, $\Pi (X,\hat{X})$ denotes the set of all transport plans between each cell of **X** and $\hat{X}$ and *D_ij_* represents the $\ell_{2}$ distance, such that the Wasserstein metric adopts the minimal-cost transport plan Γ to measure the data dissimilarity.

### 2.3 scNODE uses dynamic regularization for learning a robust latent space

The VAE and its corresponding latent space are trained using training (or observed) timepoints. In the temporal scRNA-seq data where gene expression distribution changes substantially, a fixed low-dimensional representation obtained using training timepoints may not generalize to a testing (or unobserved) timepoint. To overcome this distribution shift issue, scNODE uses a **dynamic regularization** term to update the VAE space dynamically such that it captures both cellular variations and the developmental dynamics of the scRNA-seq data. Specifically, scNODE minimizes the difference between the latent variables generated by the VAE (i.e. ${\text{Enc}}_{\phi }(X)$) and the dynamics learned by the ODE (i.e. **Z**). Because we have no correspondence between them, scNODE again uses Wasserstein distance to evaluate their difference at each observed timepoint t∈T and defines the dynamic regularization as

(6) $$\begin{array}{c} R(T)=\sum_{t\in T}\;\text{Wass}\left(Z_{\text{enc}}^{\left(t\right)},\;Z^{\left(t\right)}\right)\;\text{with}\;\\ Z_{\text{enc}}^{\left(t\right)}∼N(\mu_{t},\sigma_{t})\;\text{and}\;\mu_{t},\sigma_{t}={\text{Enc}}_{\phi }(X^{\left(t\right)}),\\ Z^{\left(t\right)}\;\text{comes from neural ODE Eq}.4 \end{array}$$

Therefore, scNODE jointly updates VAE and neural ODE, optimizing parameters ϕ, *θ*, and *ω*, by minimizing the regularized loss function

(7) $$\begin{array}{c} L_{\text{dyn}}=\sum_{t\in T}\;\text{Wass}\left(X^{\left(t\right)},\;{\hat{X}}^{\left(t\right)}\right)+\beta R(T), \end{array}$$

so that the overall dynamics update the final latent space of scNODE through dynamic regularization and corresponding hyperparameter *β*.

Our dynamic regularization improves upon previous generative models (Schiebinger *et al.* 2019, Tong *et al.* 2020, Yeo *et al.* 2021, Huguet *et al.* 2022), which fix the latent space at the beginning and then learn the cell dynamics. scNODE uses the dynamic manifold learned from neural ODE as a prior and enforces VAE latent space to incorporate it using dynamic regularization. Therefore, updating the VAE ensures that scNODE fits the data better and learns a latent space that is robust to distribution shift when generating gene expression for unobserved timepoints. Previous work by Connor *et al.* (2021) similarly introduced a linear dynamic system-based model to learn a smooth low-dimensional manifold and then incorporated this manifold (as a prior) into VAE to encourage the latent space to fit the data manifold better. This resolved the issue that VAE latent representations sometimes would not match the true data manifold and poorly defined natural paths between data points. Therefore, our dynamic regularization will result in improved representation learning.

## 3 Results

### 3.1 Experimental setup

#### 3.1.1 Datasets

We use three publicly available scRNA-seq datasets to demonstrate the capabilities of scNODE in predicting developmental dynamics from real-world single-cell gene expression data. These datasets are summarized in Table 1, have >10 timepoints, and cover various species and tissues. For each timepoint, gene expression is measured at a developmental stage (ZB), every hour (DR), or every 12 h (SC). To make computations tractable, we relabel timepoints with consecutive natural numbers starting from 0. In particular, the meaning of interpolating or extrapolating is defined by these data-specific units. For example, extrapolating one timepoint in the DR dataset means predicting gene expressions for the next hour. We use the data after removing batch effects among different timepoints. In each experiment, we select the top 2000 most HVGs from the datasets and normalize the unique molecular identifier count expression through a log transformation with pseudo-count.

**Table 1.** Data descriptions of the three real-world scRNA-seq datasets used in experiments.

ID	Dataset	Species	No. of cells	No. of timepoints	Source
ZB	zebrafish embryo	*Danio rerio*	38 731	12	Broad Single-Cell Portal SCP162 (Farrell *et al*. 2018)
DR	drosophila	*Drosophila melanogaster*	27 386	11	GSE190149 (Calderon *et al*. 2022)
SC	Schiebinger2019	*Mus musculus*	236 285	19	Schiebinger *et al*. (2019)

#### 3.1.2 Training and testing

We test scNODE’s performance in predicting gene expression at unobserved timepoints. Specifically, for each dataset, we remove several timepoints to test whether scNODE can recover these left-out observations. We design three tasks: (i) **easy** tasks where we remove uniformly spaced timepoints in the middle of the measured time range for interpolating, (ii) **medium** task where we remove the last few timepoints for extrapolating beyond the measured time range, and (iii) **hard** tasks where we combine the interpolation and extrapolation schemes. Supplementary Table S1 shows the timepoints removed in each task. We consider the left-out timepoints to be the testing set, and the remaining timepoints are used to train the model. In each case, HVGs are selected based on cells corresponding to the training timepoints in order to avoid data leakage.

#### 3.1.3 Baselines

We compare scNODE with two main state-of-the-art generative methods that are capable of both interpolating and extrapolating gene expression:

- **PRESCIENT**: Yeo *et al.* (2021) propose a generative model, called Potential eneRgy undErlying Single Cell gradIENTs (PRESCIENT), to learn the differentiation landscape from single-cell time-series gene expression data. PRESCIENT maps single-cell gene expression to a lower-dimensional PCA space and models cell differentiation with a neural ODE.
- **MIOFlow**: Huguet *et al.* (2022) integrate geodesic VAE and neural ODE to model the paths of cells in a lower-dimensional latent space while cellular variations are preserved.

#### 3.1.4 Hyperparameter tuning

In each task for every dataset, we select corresponding hyperparameters for all methods (our and baselines) that yield the minimum averaged Wasserstein distance using the 3-fold cross-validation scheme. We use Optuna (Akiba *et al.* 2019) to automatically determine the optimal hyperparameters and use sufficiently large hyperparameter ranges for search and evaluation. The hyperparameter ranges of scNODE and baselines are listed in Supplementary Table S2. We set the latent space dimension as 50, use the first-order Euler ODE solver, and set ODE step size Δt=0.1 for all methods, and run each method for sufficient iterations to ensure they converge. We let every model predict 2000 cells at each testing timepoint to ensure a fair metric comparison. Moreover, we evaluate scNODE performance using different hyperparameter settings, conduct ablation studies, and give heuristic guidance on how to set hyperparameters in real-world scenarios in Supplementary Section S6.3.

### 3.2 scNODE can accurately predict gene expression at unobserved timepoints

**Figure 2.**
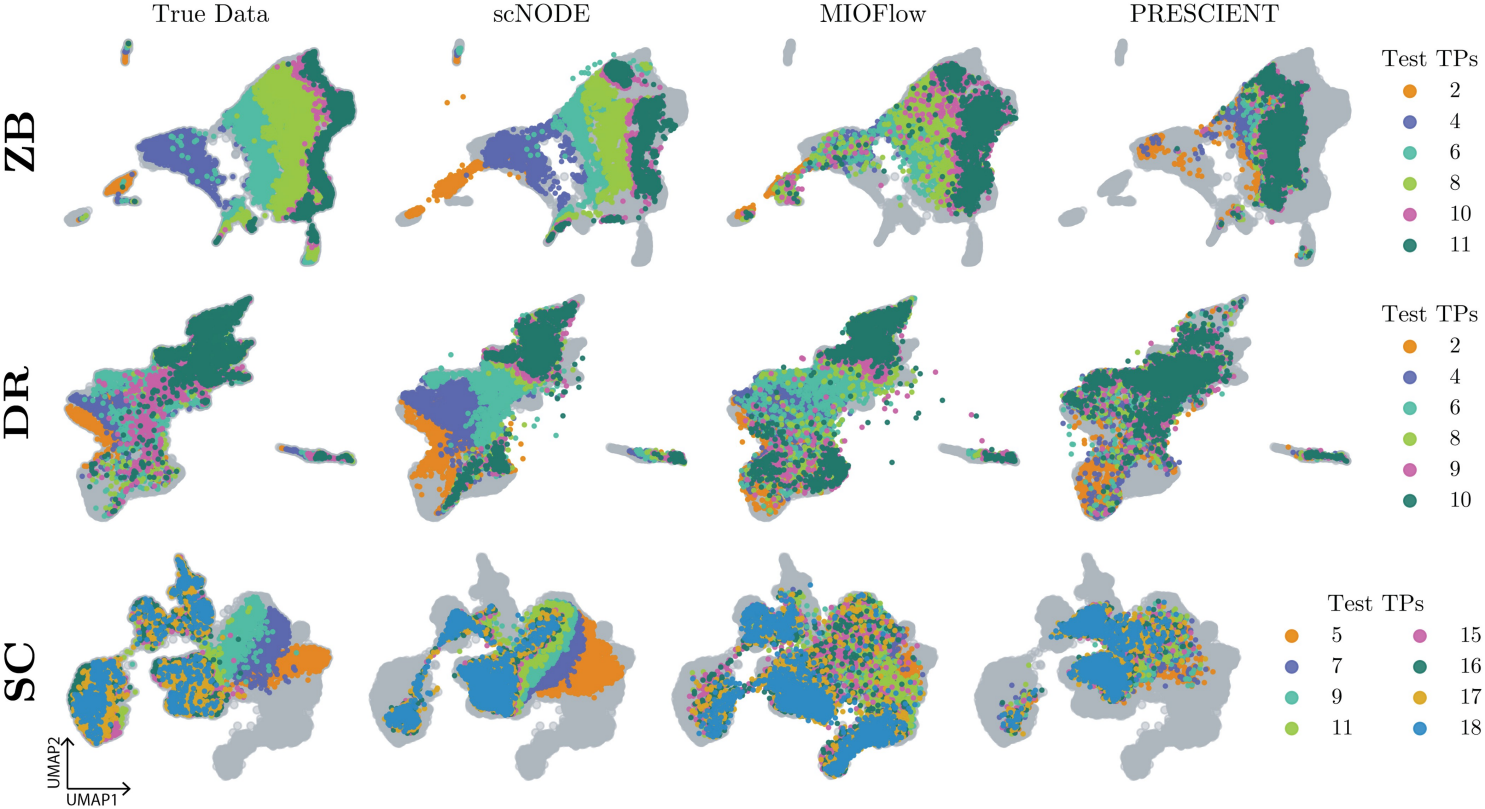
2D UMAP visualization of true and predicted gene expressions in hard tasks. Gray points represent training data.

We compare scNODE’s performance with baseline methods for predicting gene expression for left-out timepoints. Figure 2 (along with Supplementary Figs. S1–S3) visualizes true gene expression values and model predictions for left-out testing timepoints in 2D Uniform Manifold Approximation and Projection (UMAP) (McInnes *et al.* 2018) space. Our results indicate scNODE’s predictions align well with ground truth. Qualitative evaluation of all scRNA-seq datasets indicates that scNODE can accurately predict gene expression at missing timepoints.

We then quantitatively evaluate model predictions. We use the Wasserstein distance to measure the similarity between true and predicted gene expression at each testing timepoint, where a lower value corresponds to more accurate predictions. This evaluation metric has been used in previous studies (Tong *et al.* 2020, Yeo *et al.* 2021) because Wasserstein distance learns a soft mapping across the distributions, and we do not have one-to-one cell matching between true and predicted gene expression for single-cell measurements. Table 2 shows the Wasserstein distance of left-out testing timepoints averaged on five trials for defined hard tasks, and Supplementary Table S3 shows metrics for easy and medium tasks. scNODE clearly outperforms the baselines in most cases. In other cases where scNODE has the second best predictions, such as *t *=* *7 of SC hard task, scNODE has similar performance as MIOFlow but significantly performs better than PRESCIENT. Moreover, in medium and hard tasks, where extrapolations are required, scNODE can have substantial improvement over baselines. For example, at *t *=* *15 of SC hard task (in Table 2), Wasserstein distance of scNODE predictions is around 132, while that of PRESCIENT and MIOFlow are about 150 and 162 respectively. In Supplementary Fig. S4, we further evaluate all model predictions when extrapolating more timepoints. As expected, their extrapolations become less accurate the farther out predictions are made. But scNODE still demonstrates improvement in most cases. Therefore, scNODE has consistent good performance, especially in more challenging prediction tasks.

**Table 2.** Wasserstein distance of predictions in hard tasks (inter- and extrapolation).^a^

Method	ZB/hard task					
	Interpolation				Extrapolation	
	*t *=* *2	*t *=* *4	*t *=* *6	*t *=* *8	*t *=* *10	*t *=* *11
scNODE	**579.10**	**508.55**	**440.92**	**517.81**	**652.36**	**707.10**
MIOFlow	580.18	516.59	453.61	536.35	671.23	734.42
PRESCIENT	1381.96	1002.62	730.974	701.29	916.51	973.17

Method	DR/hard task					
	Interpolation			Extrapolation		
	*t *=* *2	*t *=* *4	*t *=* *6	*t *=* *8	*t *=* *9	*t *=* *10
scNODE	445.82	**464.78**	535.78	**600.18**	585.60	**718.20**
MIOFlow	**443.56**	469.51	**532.93**	617.48	680.41	852.02
PRESCIENT	524.38	511.61	539.38	621.31	**575.45**	718.56

Method	SC/hard task							
	Interpolation				Extrapolation			
	*t *=* *5	*t *=* *7	*t *=* *9	*t *=* *11	*t *=* *15	*t *=* *16	*t *=* *17	*t *=* *18
scNODE	55.22	**59.89**	**103.26**	**140.81**	**132.86**	**148.89**	**137.90**	**151.13**
MIOFlow	**55.07**	61.80	108.72	156.51	162.12	191.40	189.39	215.74
PRESCIENT	85.36	87.47	114.16	142.03	150.53	161.59	147.23	155.06

a **Bold** numbers denote the best prediction, and underlined numbers represent the second best.

**Figure 3.**
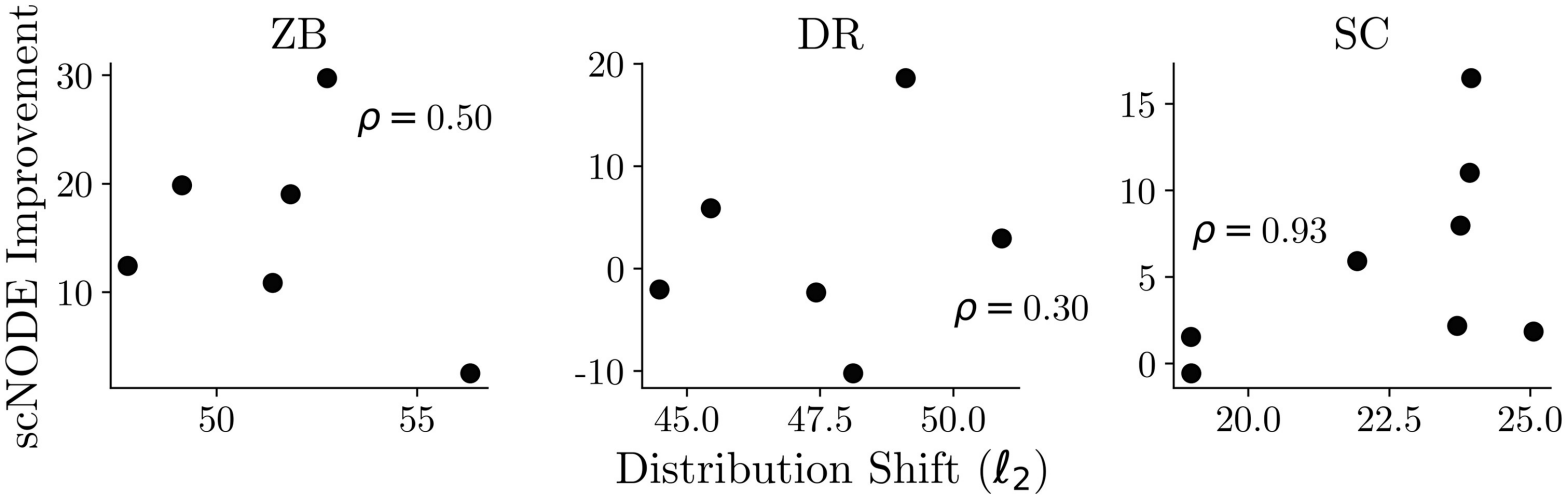
scNODE improvements over baseline models versus distribution shift levels of testing points on hard tasks. Each point denotes one testing timepoint with scNODE improvements averaged on five trials. *ρ*: Spearman’s *ρ* correlations between improvements and distribution shift.

Next, we validate that scNODE is more robust to distribution shift when testing timepoints have substantially different distributions from training data compared to baseline methods. Specifically, for the hard task of all three datasets, we first compute the distribution shift level for each testing timepoint as the averaged pairwise $\ell_{2}$ distance between cells from training and testing timepoints. Here, a higher value indicates the testing point is more different from the training data. Then, we define scNODE’s improvement as the difference between its performance (calculated as Wasserstein distance between model predictions and ground truth) and the performance of the best baseline model. Thus, a higher value indicates that scNODE improves the predictions over the baseline. Figure 3 shows that scNODE improvements positively correlate with the distribution shift level (Spearman correlation ρ≥0.3 and is as high as 0.93.), implying scNODE achieves more improvements for significant distribution shifts. In addition, Supplementary Fig. S4 shows that scNODE’s performance is more stable than baselines when extrapolating multiple timepoints. The ablation study in Supplementary Table S6 further shows that excluding dynamic regularization worsens scNODE predictions. Therefore, due to the dynamic regularization introduced in our framework, scNODE obtains significant improvements over baseline models when distribution shifts of testing timepoints are large from the training timepoints.

### 3.3 scNODE predictions for unobserved timepoints help recover cell trajectories

**Figure 4.**
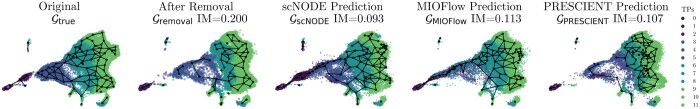
Cell trajectories of ZB data with all timepoints, after removal of timepoints in the hard task, and with model predictions. The connective structure is constructed with PAGA, where black nodes represent cell clusters and edges connect two nodes if their expressions are similar. We show the IM index between $G_{\text{true}}$ and the corresponding graph.

To determine if scNODE can aid with temporal downstream analysis, we carry out trajectory analysis on both real and predicted single-cell gene expression. Specifically, we focus on the hard task of all three datasets. Figure 4 visualizes the ZB data with all timepoints, after the removal of testing timepoints, and with predictions from all models. We apply partition-based graph abstraction (PAGA) (Wolf *et al.* 2019), a method that computes the topological structures of cellular populations and has been used in previous studies (Luecken and Theis 2019, Saelens *et al.* 2019, Han *et al.* 2020) to understand cell developmental topologies. We find that after timepoint removal, the topology breaks down due to the gaps between timepoints, which can impede the trajectory analysis, while scNODE predictions recover smooth and continuous trajectories. To quantitatively compare scNODE with other models in helping infer cell trajectories, we use the Ipsen-Mikhailov (IM) distance (Ipsen 2004, Saelens *et al.* 2019) to measure the similarity between the cell trajectory graphs constructed in each case. $\text{IM}(G_{1},G_{2})$ is a graph similarity measurement defined as the square-root difference between the Laplacian spectrum of graphs $G_{1}$ and $G_{2}$. It ranges from 0 to 1, where 0 indicates maximum similarity between two graph structures and 1 indicates maximum dissimilarity. We find $\text{IM}(G_{\text{true}},G_{\text{removal}})=0.200$ and $\text{IM}(G_{\text{true}},G_{\text{scNODE}})=0.093$, indicating $G_{\text{scNODE}}$ is more similar to $G_{\text{true}}$ than $G_{\text{removal}}$ such that scNODE predictions help recovering cell trajectories. Moreover, $\text{IM}(G_{\text{true}},G_{\text{scNODE}})$ being smaller than $\text{IM}(G_{\text{true}},G_{\text{MIOFlow}})$ and $\text{IM}(G_{\text{true}},G_{\text{PRESCIENT}})$ (Fig. 4) implies that scNODE predictions for missing timepoints best help to infer cell trajectories. We observe same trend for DR and SC datasets as well, where IM distance is lowest for scNODE (Supplementary Fig. S8).

### 3.4 scNODE’s interpretable latent space assists with perturbation analysis

scNODE’s ability to learn an informative and robust latent space allows it to detect key driver genes for cell developmental paths and simulate cells with perturbed gene expression. This ability can be useful for performing *in silico* perturbation predictions. We validate this hypothesis by using the differential dynamics learned from the ZB dataset. We choose ZB data because the original dataset provides cell type labels at the last timepoint, which enables us to evaluate the perturbation predictions.

**Figure 5.**
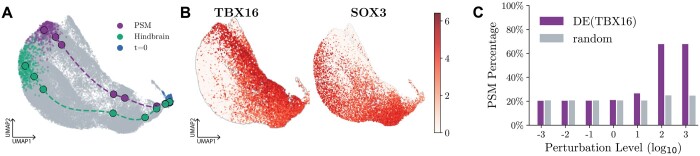
scNODE perturbation analysis results. (A) 2D UMAP visualization of the least action path between cells at the starting point (*t *=* *0) and the PSM and Hindbrain cell populations, respectively. (B) Gene expression value of the top-rank DE gene of PSM path (TBX16) and Hindbrain path (SOX3). (C) The ratio of PSM cells in predictions of different perturbation levels when perturbing DE genes and random non-DE genes.

We first train scNODE with all timepoints in the ZB dataset and map all cells to the latent space with the learned scNODE encoder. In the latent space, we construct a most probable path between any two points through the Least Action Path (LAP) method (Qiu *et al.* 2012, 2022, Wang *et al.* 2014), which finds the optimal path between two cell states while minimizing its action and transition time (see Supplementary Section S6.5). Here, we construct LAP paths from cells at the starting point (i.e. *t *=* *0) to presomitic mesoderm (PSM) and Hindbrain cell populations (Fig. 5A).

Once the optimal path is constructed, we use the Wilcoxon rank-sum test to find differentially expressed (DE) genes for each path, representing key driver genes for the developmental trajectories. Figure 5 shows the expression of top-rank DE genes for the PSM path (TBX16) and Hindbrain path (SOX3), such that their respective expression levels vary along the path (as well as in adjacent regions). Warga *et al.* (2013) have found TBX16 regulates intermediate mesoderm cell fate. Other studies (Vriz *et al.* 1996, Dee *et al.* 2008) have found SOX3 regulates neural fates and is involved in central nervous system development.

Finally, we perturb the expression profiles of key genes in all cells at the starting timepoint by multiplying their expression values with different levels of coefficient $\left\{10^{-3},\ldots ,10^{3}\right\}$ to mimic overexpressing and knocking-out, and let scNODE predict trajectories for the perturbed gene expression. The perturbations are expected to result in changes in cell fates. We classify predicted cells with a Random Forest (RF) classifier (Breiman 2001) trained with all unperturbed cells. We find overexpressing TBX16 expression results in the increment of PSM cell ratios (Fig. 5C) to over 70%, while perturbing random non-DE genes leads to no changes in cell ratios. Moreover, the ratio of Hindbrain cells decreased by around 25% when we overexpressed SOX3 expressions (Supplementary Fig. S9). These results indicate that scNODE learns an interpretable latent space, where we can detect development-related DE genes and conduct *in silico* perturbation analysis.

## 4 Conclusion

We propose a generative model called scNODE to predict gene expression during cell development. Given a time-series scRNA-seq dataset, our model can both interpolate and extrapolate gene expression. Predicting gene expression for unobserved timepoints enables critical downstream analyses, such as differential gene expression analysis, perturbation analysis, and trajectory inference. To make accurate predictions, scNODE uses a nonlinear dimensionality reduction approach, neural ODE, and dynamic regularization. The regularization improves the robustness of the learned latent representation to distribution shifts in unobserved timepoints, resulting in better predictive performance.

We find that the primary challenge for the temporal scRNA-seq prediction task is extrapolating to multiple timepoints beyond the last observed timepoint, as the accuracy deteriorates the farther timepoint predictions. Moreover, time units in predictions are defined by time intervals in the data. For example, a generative model cannot accurately produce gene expression for every hour if the model is trained on observations that are only measured every day, especially when the development is rapid or contains complex trajectories.

In future work, we will incorporate knowledge about cell proliferation—which is known to be important to cellular differentiation—into the model to improve extrapolating performance. Also, although RNA velocity methods can be used as complementary approaches to scNODE to learn fine-grained dynamics from coarse-grained timepoints. We will also incorporate other modalities, such as single-cell chromatin accessibility (scATAC) datasets, which exhibit a regulatory mechanism that scRNA-seq data might not capture.

## Supplementary Material

btae393_Supplementary_Data

## Data Availability

All data used in this study are available at https://github.com/rsinghlab/scNODE.
