# Supplementary material for: scNODE : generative model for temporal single cell transcriptomic data prediction
Source: Bioinformatics. 2024 Sep 4;40(Suppl 2):ii146–54. doi: 10.1093/bioinformatics/btae393 (PMC11373355; doi:10.1093/bioinformatics/btae393)
Supplement: btae393_Supplementary_Data [file btae393_supplementary_data.zip › scNODE_ECCB.pdf]

Journal Title Here, 2022, 1–9
doi: DOI HERE
Advance Access Publication Date: Day Month Year
Paper

scNODE : Generative Model for Temporal Single Cell Transcriptomic Data Prediction

Jiaqi Zhang,[1] Erica Larschan,[2,3] Jeremy Bigness[2,*] and Ritambhara Singh[1,2,*]

[1]Department of Computer Science, Brown University, 115 Waterman St., 02906, RI, United States, [2]Center for Computational Molecular Biology, Brown University, 164 Angell St., 02912, RI, United States and [3]Department of Molecular Biology, Cell Biology and Biochemistry, Brown University, 185 Meeting St., 02912, RI, United States

[*]Corresponding authors. {jeremy_bigness, ritambhara}@brown.edu

FOR PUBLISHER ONLY Received on Date Month Year; revised on Date Month Year; accepted on Date Month Year

Abstract

Measurement of single-cell gene expression at different timepoints enables the study of cell development. However, due to the resource constraints and technical challenges associated with the single-cell experiments, researchers can only profile gene expression at discrete and sparsely-sampled timepoints. This missing timepoint information impedes downstream cell developmental analyses. We propose scNODE, an end-to-end deep learning model that can predict *in silico* single-cell gene expression at unobserved timepoints. scNODE integrates a variational autoencoder (VAE) with neural ordinary differential equations (ODEs) to predict gene expression using a continuous and non-linear latent space. Importantly, we incorporate a dynamic regularization term to learn a latent space that is robust against distribution shifts when predicting single-cell gene expression at unobserved timepoints. Our evaluations on three real-world scRNA-seq datasets show that scNODE achieves higher predictive performance than state-of-the-art methods. We further demonstrate that scNODE's predictions help cell trajectory inference under the missing timepoint paradigm and the learned latent space is useful for *in silico* perturbation analysis of relevant genes along a developmental cell path. The data and code are publicly available at https://github.com/rsinghlab/scNODE.

Key words: scRNA-seq, generative model, gene expression prediction

1. Introduction

A fundamental challenge in biology is understanding how gene expression changes over time during cell development (Moris et al., 2016; Fleck et al., 2022). With the advent of high-throughput single-cell RNA sequencing (scRNA-seq), researchers now routinely profile gene expression at single-cell resolution (Hwang et al., 2018; Chen et al., 2019), revealing substantial heterogeneity within the same tissue type or developmental stage (Chen et al., 2022a; Ding et al., 2022). Profiling scRNA-seq data at multiple timepoints allows us to understand how biological processes unfold within cell populations by inferring cellular trajectories, identifying cell fate transitions, and characterizing differentially expressed genes (Qiu et al., 2022; Liu et al., 2022).

Temporal scRNA-seq experiments, however, have significant limitations that impede developmental studies. Due to the substantial time and resources involved in conducting these experiments, researchers generally only profile gene expression at discrete and sparsely-sampled timepoints (Ding et al., 2022). Since it is infeasible to make continuous-time observations, this results in information loss between consecutively measured discrete timepoints. Therefore, it is crucial to develop a model that predicts realistic *in silico* gene expression at any timepoint, whether between (interpolation) or beyond the measured time intervals (extrapolation), to enable single-cell temporal analyses (Fig. 1**A-B**).

Single-cell gene expression prediction has been investigated in previous studies (Cao et al., 2021) through various generative models. Existing methods use generative adversarial networks (GANs) (Marouf et al., 2020) or statistical frameworks (Zappia et al., 2017; Risso et al., 2018; Li and Li, 2019; Sun et al., 2021) to fit characteristics

© The Author 2022. Published by Oxford University Press. All rights reserved. For permissions, please e-mail: journals.permissions@oup.com

of the observed single-cell gene expression and augment the dataset. However, these methods are designed for single-cell gene expression measured at a single time snapshot and do not consider developmental dynamics.

Several computational methods have been developed to learn gene expression dynamics by inferring cell trajectories (Trapnell et al., 2014; Qiu et al., 2017; Herman et al., 2018; Saelens et al., 2019; Wolf et al., 2019; Li, 2023) from temporal scRNA-seq. However, most of them infer pseudo-times, which analyze cell differentiation based on geometric distances in low-dimensional spaces. Hence, they do not accurately model how cells develop in real physical time. Sagittarius method (Woicik et al., 2023) predicts gene expressions at future timepoints but requires cell matching between timepoints. This matching is hard to obtain for real-world scRNA-seq as the cells are lysed during the experiment. RNA velocity based methods (La Manno et al., 2018; Bergen et al., 2021, 2020) are used to uncover developmental trends and underlying kinetics of gene expression. These methods require deeply sequenced datasets to obtain spliced and unspliced counts as prior information, and most of them are non-generative methods. Waddington-OT (Schiebinger et al., 2019) and TrajectoryNet (Tong et al., 2020) interpolate single-cell gene expression between two timepoints in a developmental trajectory but are unable to extrapolate beyond the observed timepoints.

Recently, a few generative methods have modeled cell differentiation trajectory and predicted gene expression at multiple unobserved time points. For example, PRESCIENT (Yeo et al., 2021) applies principal component analysis (PCA) to reduce high-dimensional gene space to a low-dimensional representation. It then applies neural ordinary differential equations (ODEs) (Chen et al., 2018) in this low-dimensional space to model the cell developmental trajectory. Multiple studies (Ding et al., 2018; Xiang et al., 2021; Tran et al., 2021) have found that PCA-based low-dimensional representations of scRNA-seq expression cannot capture complex relationships in the highly heterogeneous single-cell data. Thus, PCA may have the issue of overcrowding representation (Tran et al., 2021; Kobak and Berens, 2019), where cells of different types are poorly separated in PCA-based low-dimensional space, and cellular variations are lost. MIOFlow (Huguet et al., 2022) replaces PCA with a geodesic variational autoencoder (VAE) to learn non-linear low-dimensional representations that better retain cellular variations. However, this method fixes its low-dimensional space at the beginning, followed by neural ODE modeling. We hypothesize that a fixed low-dimensional representation obtained using observed timepoints may not generalize to an unobserved timepoint, if its underlying distribution differs substantially.

We propose a single-cell neural ODE (`scNODE`) framework, which integrates a VAE (Kingma et al., 2019) with neural ODE to predict accurate gene expression at any unmeasured timepoint using a dynamic low-dimensional space (Fig. 1**C**). First, `scNODE` uses VAE to obtain a low-dimensional representation that retains cellular variations. `scNODE` then predicts single-cell gene expression for unmeasured timepoints with a neural ODE. Importantly, `scNODE` uses a *dynamic regularization* term to incorporate the dynamic manifold learned from neural ODE as a prior. Therefore, the learned VAE space is robust to the distribution shift between observed and unobserved time points. We evaluate `scNODE` on three real-world scRNA-seq datasets. Our results demonstrate that `scNODE` predicts single-cell gene expression more accurately than state-of-the-art methods for unobserved timepoints (interpolation and extrapolation). We further show that `scNODE` gene expression predictions assist with cell developmental trajectory inference. Finally, `scNODE` learns an interpretable low-dimensional space, which enables conducting *in silico* perturbation analysis of relevant genes to study cell development.

2. Method

Notation: Let $\mathbf{X}^{(t)} \in \mathbb{R}^{n_t \times p}$ denote the gene expression matrix at the t-th time point of n_t cells by p genes. Given gene expression measurements at observed timepoints $\{\mathbf{X}^{(t)}\}_{t\in\mathcal{T}}$, our goal is to predict gene expression at any timepoint. Here, $\mathcal{T} \subseteq \{0, 1, 2, \cdots\}$ denotes the observed timepoint indices. Note that we assume the same set of highly variable genes (HVGs) is measured at each time point, but due to the destruction of cells during scRNA experiments, a different set of cells is measured at each time point. Furthermore, let $\mathbf{Z}^{(t)} \in \mathbb{R}^{n_t \times d}$ denote the d-dimensional ($d \ll p$) latent variable at the t-th time point.

2.1. `scNODE` uses VAE to learn complex low-dimensional space

`scNODE` uses a Variational Autoencoder (VAE) to learn a low-dimensional representation (also known as latent space) of the single-cell gene expression measurements at the observed timepoints. A VAE is a neural network-based generative model that maps high-dimensional data to a low-dimensional representation and is widely used in many single-cell studies (Grønbech et al., 2020; Svensson et al., 2020; Wang and Gu, 2018; Lin et al., 2020), showing superior performance. The benefit of using VAEs for single-cell data over PCA is that the non-linearity of neural networks can more effectively capture complex cell relationships and cellular variations.

`scNODE` first pre-trains a VAE to obtain a latent space that captures the gene expression of single cells at all available training timepoints. Specifically, `scNODE` trains the VAE component to perform multi-timepoint modeling by inputting the gene expression of all observed cells $\mathbf{X}_{\text{ALL}} = \text{CONCAT}\left(\mathbf{X}^{(t)} \mid t \in \mathcal{T}\right)$. VAE is composed of two neural networks: (1) the encoder network Enc_ϕ maps expression from gene space $\mathbb{R}^p$ to a low-dimensional latent space $\mathbb{R}^d$, parameterized by a Gaussian distribution $\mathcal{N}(\mu, \sigma)$, and (2) the decoder network Dec_θ maps samples in the latent space to gene space in the opposite direction to reconstruct the input. Given $\mathbf{X}_{\text{ALL}}$, VAE learns latent

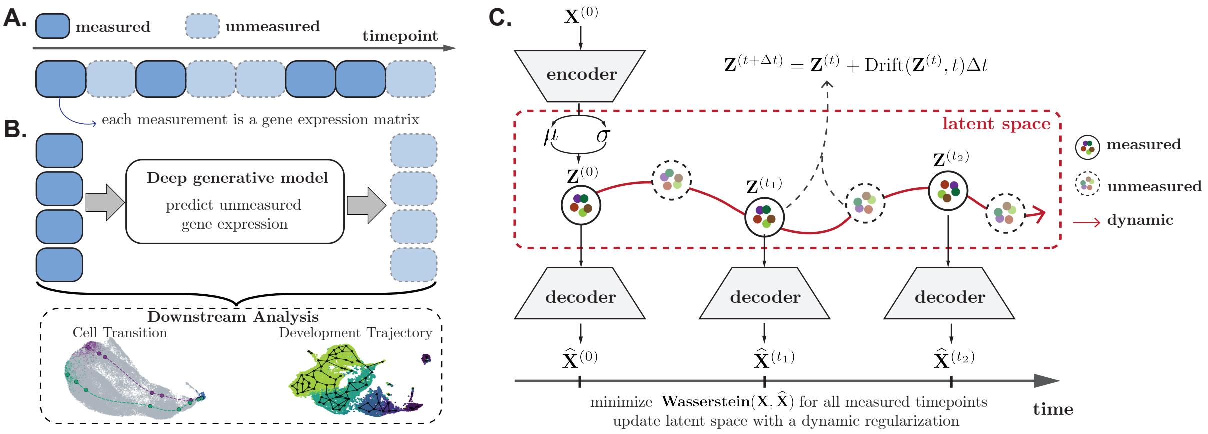

Fig. 1: **Model overview.** (**A**) The measurements in temporally recorded single-cell data are sparse and unaligned, such that some timepoints are not measured and measurements at different timepoints contain different sets of cells. The data sets in this study sample cellular populations at uniformly spaced time points, although uniform spacing is not a requirement (as depicted in this graphic). (**B**) `scNODE` is used to predict expression at unmeasured timepoints. Predictions can be used to analyze cell state transitions and developmental trajectories. (**C**) `scNODE` uses VAEs to find an optimal low-dimensional representation. `scNODE` then uses this representation to solve neural ODEs and predict gene expression at any timepoint $t = t_1, t_2, \cdots$ starting from the first timepoint $t = 0$. Optimal models are fitted by minimizing the Wasserstein distance between observations and predictions at measured timepoints. `scNODE` also introduces a dynamic regularization to learn a latent space robust to distribution shift.

representations through

$$\begin{aligned} \mu_{\text{ALL}}, \sigma_{\text{ALL}} &= \text{Enc}_\phi(\mathbf{X}_{\text{ALL}}), \\ \mathbf{Z}_{\text{ALL}} &\sim \mathcal{N}(\mu_{\text{ALL}}, \sigma_{\text{ALL}}), \\ \widehat{\mathbf{X}}_{\text{ALL}} &= \text{Dec}_\theta(\mathbf{Z}_{\text{ALL}}). \end{aligned} \tag{1}$$

The encoder and decoder networks are parameterized by ϕ and θ, correspondingly. VAE minimizes a combination of the (1) mean squared error (MSE) between input gene expression and the reconstructed gene expression from the decoder and (2) the Kulback-Leibler (KL) divergence (Csiszár, 1975) between the latent distribution and a standard Gaussian prior:

$$\mathcal{L}_{\text{pre}} = \text{MSE}\left(\mathbf{X}_{\text{ALL}},\ \widehat{\mathbf{X}}_{\text{ALL}}\right) + \lambda \text{KL}\left(\mathcal{N}(\mu_{\text{ALL}}, \sigma_{\text{ALL}}),\ \mathcal{N}(0,1)\right). \tag{2}$$

KL divergence is an information-theoretic quantity that quantifies the distance between two probability distributions. By using a Gaussian prior, the KL divergence forces the encoder to find latent representations of the cells that are well-separated. `scNODE` pre-trains the VAE component with Eq. 2, such that the encoder latent space preserves the variation of all observed cells. Next, we model the developmental dynamics of this latent space.

2.2. `scNODE` uses neural ODE to model cell dynamics

`scNODE` uses ordinary differential equations (ODEs) to model cell developmental dynamics in the latent space learned by the VAE. An ODE is an equation describing how a quantity x changes with respect to an independent variable y, such that $\mathrm{d}x = f(x; y)\mathrm{d}y$ where function f represents the derivative. Therefore, we can use differential equations to model how gene expression changes with respect to time. However, for the high-dimensional data, finding the solution of the derivative function f through numerical methods is intractable and computationally expensive (Kidger, 2022). Therefore, recent studies adopt neural networks to approximate the derivative function and have proposed neural ODEs (Chen et al., 2018). Neural ODEs (formulated with respect to time) are useful for constructing continuous-time trajectories and have been explored before to model single-cell development (Matsumoto et al., 2017; Qiu et al., 2022; Chen et al., 2022b).

`scNODE` uses neural ODEs to parameterize the continuous dynamics of gene expression in the latent space. Specifically, `scNODE` quantifies changes of cell latent representation $\mathbf{Z}^{(t)}$ (from VAE encoding) at time t through a neural ODE

$$\mathrm{d}\mathbf{Z}^{(t)} = \text{Drift}_\omega\left(\mathbf{Z}^{(t)}; t\right) \cdot \mathrm{d}t \tag{3}$$

Here, Drift_ω is a non-linear neural network with parameterization ω, modeling the developmental velocities in the latent space, such that $\text{Drift}_\omega(\mathbf{Z}^{(t)}; t)$ represents the direction and strength of cellular transitions. `scNODE` computes the latent representation $\mathbf{Z}^{(0)}$ of cells at the first timepoint $t = 0$ through the encoder network (pre-trained in the previous step) and predicts the subsequent cell states step-wise at any timepoint t as follows:

$$\begin{aligned} \mathbf{Z}^{(0)} \sim \mathcal{N}(\mu_0, \sigma_0) \quad \text{and} \quad \mu_0, \sigma_0 = \text{Enc}_\phi(\mathbf{X}^{(0)}), \\ \mathbf{Z}^{(t+\Delta t)} = \mathbf{Z}^{(t)} + \text{Drift}_\omega\left(\mathbf{Z}^{(t)}, t\right)\Delta t. \end{aligned} \tag{4}$$

Here, hyperparameter Δt denotes step size and drift term $\text{Drift}_\omega\left(\mathbf{Z}^{(t)}, t\right)\Delta t$ represents the forward steps taken in the latent space. While there are several methods that can solve this ODE, in this work, we apply the commonly

used first-order Euler method (in Eq. 4) for convenience of explanation. However, in our implementation, one can specify any ODE solver.

To fit the continuous trajectory (controlled by Drift_ω) to the observations, `scNODE` minimizes the difference between the input and the reconstructed gene expression. Specifically, at each measured timepoint $t \in \mathcal{T}$, `scNODE` uses the decoder Dec_θ to convert latent variables $\mathbf{Z}^{(t)}$ generated from Eq. 4 back to the gene space through $\widehat{\mathbf{X}}^{(t)} = \text{Dec}_\theta\left(\mathbf{Z}^{(t)}\right)$. Because we have no correspondence between true cells and cells generated from the ODE model, `scNODE` utilizes the Wasserstein metric (Cuturi, 2013) to measure the distance between distributions defined by ground truth $\mathbf{X}$ and predictions $\widehat{\mathbf{X}}$ as

$$\text{Wass}(\mathbf{X}, \widehat{\mathbf{X}}) = \left(\min_{\Gamma \sim \Pi(\mathbf{X}, \widehat{\mathbf{X}})} \sum_{i,j} D_{ij}^2 \Gamma_{ij} \right)^{1/2} \quad \text{with} \quad D_{ij} = \|\mathbf{X}_i - \widehat{\mathbf{X}}_j\|_2. \tag{5}$$

Here, $\Pi(\mathbf{X}, \widehat{\mathbf{X}})$ denotes the set of all transport plans between each cell of $\mathbf{X}$ and $\widehat{\mathbf{X}}$ and D_{ij} represents the ℓ_2 distance, such that the Wasserstein metric adopts the minimal-cost transport plan Γ to measure the data dissimilarity.

2.3. `scNODE` uses dynamic regularization for learning a robust latent space

The VAE and its corresponding latent space are trained using training (or observed) timepoints. In the temporal scRNA-seq data where gene expression distribution changes substantially, a fixed low-dimensional representation obtained using training timepoints may not generalize to a testing (or unobserved) timepoint. To overcome this distribution shift issue, `scNODE` uses a **dynamic regularization** term to update the VAE space dynamically such that it captures both cellular variations and the developmental dynamics of the scRNA-seq data. Specifically, `scNODE` minimizes the difference between the latent variables generated by the VAE (i.e., $\text{Enc}_\phi(\mathbf{X})$) and the dynamics learned by the ODE (i.e., $\mathbf{Z}$). Because we have no correspondence between them, `scNODE` again uses Wasserstein distance to evaluate their difference at each observed timepoint $t \in \mathcal{T}$ and defines the dynamic regularization as

$$\begin{gathered} \mathcal{R}(\mathcal{T}) = \sum_{t \in \mathcal{T}} \text{Wass}\left(\mathbf{Z}_{\text{enc}}^{(t)},\ \mathbf{Z}^{(t)}\right) \ \text{with} \\ \mathbf{Z}_{\text{enc}}^{(t)} \sim \mathcal{N}(\mu_t, \sigma_t) \text{ and } \mu_t, \sigma_t = \text{Enc}_\phi(\mathbf{X}^{(t)}), \\ \mathbf{Z}^{(t)} \text{ comes from neural ODE Eq. 4} \end{gathered} \tag{6}$$

Therefore, `scNODE` jointly updates VAE and neural ODE, optimizing parameters ϕ, θ, and ω, by minimizing the regularized loss function

$$\mathcal{L}_{\text{dyn}} = \sum_{t \in \mathcal{T}} \text{Wass}\left(\mathbf{X}^{(t)},\ \widehat{\mathbf{X}}^{(t)}\right) \ + \ \beta \mathcal{R}(\mathcal{T}), \tag{7}$$

so that the overall dynamics update the final latent space of `scNODE` through dynamic regularization and corresponding hyperparameter β.

Our dynamic regularization improves upon previous generative models (Schiebinger et al., 2019; Tong et al., 2020; Huguet et al., 2022; Yeo et al., 2021), which fix the latent space at the beginning and then learn the cell dynamics. `scNODE` uses the dynamic manifold learned from neural ODE as a prior and enforces VAE latent space to incorporate it using dynamic regularization. Therefore, updating the VAE ensures that `scNODE` fits the data better and learns a latent space that is robust to distribution shift when generating gene expression for unobserved timepoints. Previous work by Connor et al. (2021) similarly introduced a linear dynamic system-based model to learn a smooth low-dimensional manifold and then incorporated this manifold (as a prior) into VAE to encourage the latent space to fit the data manifold better. This resolved the issue that VAE latent representations sometimes would not match the true data manifold and poorly defined natural paths between data points. Therefore, our dynamic regularization will result in improved representation learning.

3. Results

3.1. Experimental Setup

Datasets We use three publicly available scRNA-seq datasets to demonstrate the capabilities of `scNODE` in predicting developmental dynamics from real-world single-cell gene expression data. These datasets are summarized in Table 1, have more than 10 timepoints, and cover various species and tissues. For each timepoint, gene expression is measured at a developmental stage (ZB), every hour (DR), or every 12 hours (SC). To make computations tractable, we relabel timepoints with consecutive natural numbers starting from 0. In particular, the meaning of interpolating or extrapolating is defined by these data-specific units. For example, extrapolating one timepoint in the DR dataset means predicting gene expressions for the next hour. We use the data after removing batch effects among different timepoints. In each experiment, we select the top 2000 most highly variable genes (HVGs) from the datasets and normalize the unique molecular identifier (UMI) count expression through a log transformation with pseudo-count.

Training and testing We test `scNODE` 's performance in predicting gene expression at unobserved timepoints. Specifically, for each dataset, we remove several timepoints to test whether `scNODE` can recover these left-out observations. We design three tasks: (1) **easy** tasks where we remove uniformly spaced timepoints in the middle of the measured time range for interpolating, (2) **medium** task where we remove the last few timepoints for extrapolating beyond the measured time range, and (3) **hard** tasks where we combine the interpolation and extrapolation schemes. Supplementary Table S1 shows the timepoints removed in each task. We consider the left-out timepoints to be the testing set, and the remaining timepoints are used to train the model. In each case, HVGs are selected based on cells corresponding to the training timepoints in order to avoid data leakage.

Table 1. Data descriptions of the three real-world scRNA-seq datasets used in experiments.

ID	Dataset	Species	# Cells	# Timepoints	Source
ZB	zebrafish embryo	*Danio rerio*	38731	12	Broad Single-Cell Portal SCP162 (Farrell et al., 2018)
DR	drosophila	*Drosophila melanogaster*	27386	11	GSE190149 (Calderon et al., 2022)
SC	Schiebinger2019	*Mus musculus*	236285	19	(Schiebinger et al., 2019)

Baselines We compare `scNODE` with two main state-of-the-art generative methods that are capable of both interpolating and extrapolating gene expression:

- **PRESCIENT**: Yeo et al. (2021) propose a generative model, called Potential eneRgy undErlying Single Cell gradIENTs (`PRESCIENT`), to learn the differentiation landscape from single-cell time-series gene expression data. `PRESCIENT` maps single-cell gene expression to a lower-dimensional PCA space and models cell differentiation with a neural ODE.
- **MIOFlow**: Huguet et al. (2022) integrate geodesic VAE and neural ODE to model the paths of cells in a lower-dimensional latent space while cellular variations are preserved.

Hyperparameter tuning In each task for every dataset, we select corresponding hyperparameters for all methods (our and baselines) that yield the minimum averaged Wasserstein distance using the 3-fold cross-validation scheme. We use Optuna (Akiba et al., 2019) to automatically determine the optimal hyperparameters and use sufficiently large hyperparameter ranges for search and evaluation. The hyperparameter ranges of `scNODE` and baselines are listed in Supplementary Table S2. We set the latent space dimension as 50, use the first-order Euler ODE solver, and set ODE step size $\Delta t = 0.1$ for all methods, and run each method for sufficient iterations to ensure they converge. We let every model predict 2000 cells at each testing timepoint to ensure a fair metric comparison. Moreover, we evaluate `scNODE` performance using different hyperparameter settings, conduct ablation studies, and give heuristic guidance on how to set hyperparameters in real-world scenarios in Supplementary Sec. S6.3.

3.2. `scNODE` can accurately predict gene expression at unobserved timepoints

We compare `scNODE` 's performance with baseline methods for predicting gene expression for left-out timepoints. Fig. 2 (along with Supplementary Fig. S1∼S3) visualizes true gene expression values and model predictions for left-out testing timepoints in 2D Uniform Manifold Approximation and Projection (UMAP) (McInnes et al., 2018) space. Our results indicate `scNODE` 's predictions align well with ground truth. Qualitative evaluation of all scRNA-seq datasets indicates that `scNODE` can accurately predict gene expression at missing timepoints.

We then quantitatively evaluate model predictions. We use the Wasserstein distance to measure the similarity between true and predicted gene expression at each testing timepoint, where a lower value corresponds to more accurate predictions. This evaluation metric has been used in previous studies (Yeo et al., 2021; Tong et al., 2020) because Wasserstein distance learns a soft mapping across the distributions, and we do not have one-to-one cell matching between true and predicted gene expression for single-cell measurements. Table 2 shows the Wasserstein distance of left-out testing timepoints averaged on five trials for defined hard tasks, and Supplementary Table S3 shows metrics for easy and medium tasks. `scNODE` clearly outperforms the baselines in most cases. In other cases where `scNODE` has the second best predictions, such as $t = 7$ of SC hard task, `scNODE` has similar performance as `MIOFlow` but significantly performs better than `PRESCIENT`. Moreover, in medium and hard tasks, where extrapolations are required, `scNODE` can have substantial improvement over baselines. For example, at $t = 15$ of SC hard task (in Table 2), Wasserstein distance of `scNODE` predictions is around 132, while that of `PRESCIENT` and `MIOFlow` are about 150 and 162 respectively. In Supplementary Fig. S4, we further evaluate all model predictions when extrapolating more timepoints. As expected, their extrapolations become less accurate the farther out predictions are made. But `scNODE` still demonstrates improvement in most cases. Therefore, `scNODE` has consistent good performance, especially in more challenging prediction tasks.

Table 2. Wasserstein distance of predictions in hard tasks (inter- and extrapolation). **Bold** numbers denote the best prediction, and underlined numbers represent the second best.

Method	ZB / Hard Task					
	Interpolation				Extrapolation	
	$t = 2$	$t = 4$	$t = 6$	$t = 8$	$t = 10$	$t = 11$
`scNODE`	**579.10**	**508.55**	**440.92**	**517.81**	**652.36**	**707.10**
`MIOFlow`	580.18	516.59	453.61	536.35	671.23	734.42
`PRESCIENT`	1381.96	1002.62	730.974	701.29	916.51	973.17

Method	DR / Hard Task					
	Interpolation			Extrapolation		
	$t = 2$	$t = 4$	$t = 6$	$t = 8$	$t = 9$	$t = 10$
`scNODE`	445.82	**464.78**	535.78	**600.18**	585.60	**718.20**
`MIOFlow`	**443.56**	469.51	**532.93**	617.48	680.41	852.02
`PRESCIENT`	524.38	511.61	539.38	621.31	**575.45**	718.56

Method	SC / Hard Task							
	Interpolation				Extrapolation			
	$t = 5$	$t = 7$	$t = 9$	$t = 11$	$t = 15$	$t = 16$	$t = 17$	$t = 18$
`scNODE`	55.22	**59.89**	**103.26**	**140.81**	**132.86**	**148.89**	**137.90**	**151.13**
`MIOFlow`	**55.07**	61.80	108.72	156.51	162.12	191.40	189.39	215.74
`PRESCIENT`	85.36	87.47	114.16	142.03	150.53	161.59	147.23	155.06

Next, we validate that `scNODE` is more robust to distribution shift when testing timepoints have substantially different distributions from training data compared to baseline methods. Specifically, for the hard task of all three datasets, we first compute the distribution shift level for each testing timepoint as the averaged pairwise ℓ_2 distance between cells from training and testing timepoints. Here, a higher value indicates the testing point is more different from the training data. Then, we

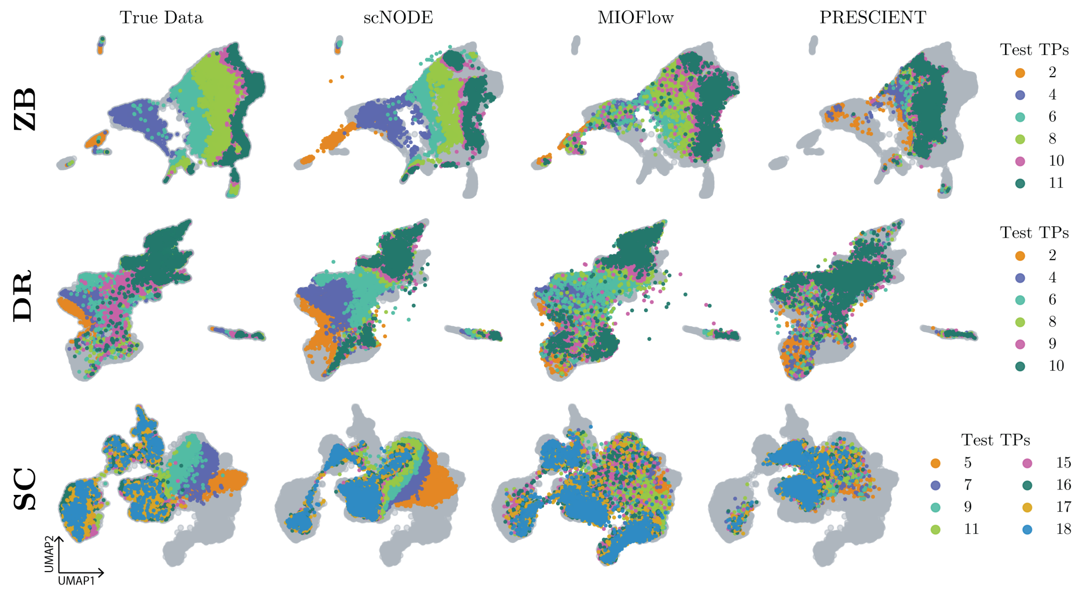

Fig. 2: 2D UMAP visualization of true and predicted gene expressions in hard tasks. Gray points represent training data.

define `scNODE` 's improvement as the difference between its performance (calculated as Wasserstein distance between model predictions and ground truth) and the performance of the best baseline model. Thus, a higher value indicates that `scNODE` improves the predictions over the baseline. Fig. 3 shows that `scNODE` improvements positively correlate with the distribution shift level (Spearman correlation $\rho \geq 0.3$ and is as high as 0.93.), implying `scNODE` achieves more improvements for significant distribution shifts. In addition, Supplementary Fig. S4 shows that `scNODE` 's performance is more stable than baselines when extrapolating multiple timepoints. The ablation study in Supplementary Table S6 further shows that excluding dynamic regularization worsens `scNODE` predictions. Therefore, due to the dynamic regularization introduced in our framework, `scNODE` obtains significant improvements over baseline models when distribution shifts of testing timepoints are large from the training timepoints.

3.3. `scNODE` predictions for unobserved timepoints help recover cell trajectories

To determine if `scNODE` can aid with temporal downstream analysis, we carry out trajectory analysis on both real and predicted single-cell gene expression. Specifically, we focus on the hard task of all three datasets. Fig. 4 visualizes the ZB data with all timepoints, after the removal of testing timepoints, and with predictions from all models. We apply partition-based graph abstraction (PAGA) (Wolf et al., 2019), a method that computes the topological structures of cellular populations and has been used in previous studies (Saelens et al., 2019; Luecken and Theis, 2019; Han et al., 2020) to understand cell developmental topologies. We find that after timepoint removal, the topology breaks down due to the gaps between timepoints, which can impede the trajectory analysis, while `scNODE` predictions recover smooth and continuous trajectories. To quantitatively compare `scNODE` with other models in helping infer cell trajectories, we use the Ipsen-Mikhailov (IM) distance (Ipsen, 2004; Saelens et al., 2019) to measure the similarity between the cell trajectory graphs constructed in each case. $\mathrm{IM}(\mathcal{G}_1, \mathcal{G}_2)$ is a graph similarity measurement defined as the square-root difference between the Laplacian spectrum of graphs $\mathcal{G}_1$ and $\mathcal{G}_2$. It ranges from 0 to 1, where 0 indicates maximum similarity between two graph structures and 1 indicates maximum dissimilarity. We find $\mathrm{IM}(\mathcal{G}_{\text{true}}, \mathcal{G}_{\text{removal}}) = 0.200$ and $\mathrm{IM}(\mathcal{G}_{\text{true}}, \mathcal{G}_{\text{scNODE}}) = 0.093$, indicating $\mathcal{G}_{\text{scNODE}}$ is more similar to $\mathcal{G}_{\text{true}}$ than $\mathcal{G}_{\text{removal}}$ such that `scNODE` predictions help recovering cell trajectories. Moreover, $\mathrm{IM}(\mathcal{G}_{\text{true}}, \mathcal{G}_{\text{scNODE}})$ being smaller than $\mathrm{IM}(\mathcal{G}_{\text{true}}, \mathcal{G}_{\text{MIOFlow}})$ and $\mathrm{IM}(\mathcal{G}_{\text{true}}, \mathcal{G}_{\text{PRESCIENT}})$ (Fig. 4) implies that `scNODE` predictions for missing timepoints best help to infer cell trajectories. We observe same trend for DR and SC datasets as well, where IM distance is lowest for `scNODE` (Supplementary Fig. S8).

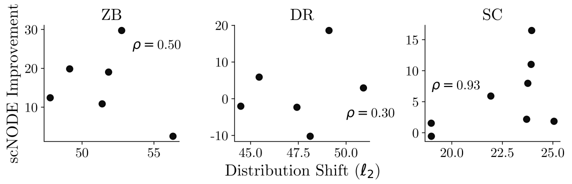

Fig. 3: `scNODE` improvements over baseline models vs. distribution shift levels of testing points on hard tasks. Each point denotes one testing timepoint with `scNODE` improvements averaged on five trials. ρ: Spearman's ρ correlations between improvements and distribution shift.

3.4. `scNODE` 's interpretable latent space assists with perturbation analysis

`scNODE` 's ability to learn an informative and robust latent space allows it to detect key driver genes for cell developmental paths and simulate cells with perturbed gene expression. This ability can be useful for performing *in silico* perturbation predictions. We validate this hypothesis by

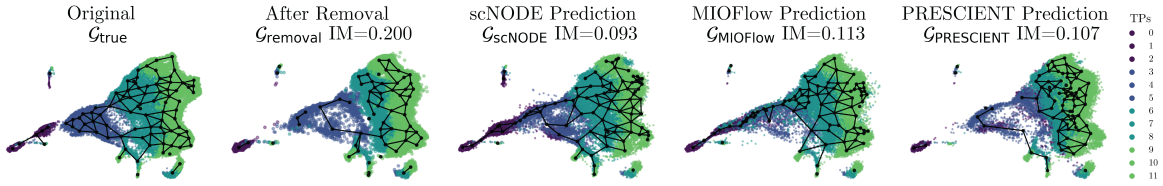

Fig. 4: Cell trajectories of ZB data with all timepoints, after removal of timepoints in the hard task, and with model predictions. The connective structure is constructed with PAGA, where black nodes represent cell clusters and edges connect two nodes if their expressions are similar. We show the IM index between $\mathcal{G}_{\mathtt{true}}$ and the corresponding graph.

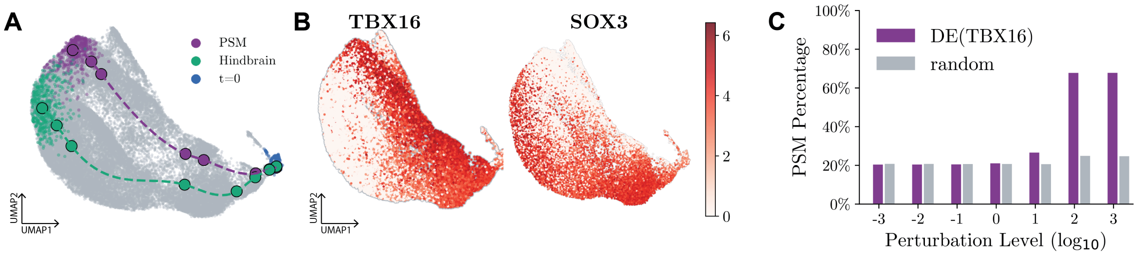

Fig. 5: `scNODE` perturbation analysis results. (**A**) 2D UMAP visualization of the least action path between cells at the starting point ($t = 0$) and the PSM and Hindbrain cell populations, respectively. (**B**) Gene expression value of the top-rank DE gene of PSM path (TBX16) and Hindbrain path (SOX3). (**C**) The ratio of PSM cells in predictions of different perturbation levels when perturbing DE genes and random non-DE genes.

using the differential dynamics learned from the ZB dataset. We choose ZB data because the original dataset provides cell type labels at the last timepoint, which enables us to evaluate the perturbation predictions.

We first train `scNODE` with all timepoints in the ZB dataset and map all cells to the latent space with the learned `scNODE` encoder. In the latent space, we construct a most probable path between any two points through the Least Action Path (LAP) method (Qiu et al., 2022, 2012; Wang et al., 2014), which finds the optimal path between two cell states while minimizing its action and transition time (see Supplementary Sec. S6.5). Here, we construct LAP paths from cells at the starting point (i.e., $t = 0$) to presomitic mesoderm (PSM) and Hindbrain cell populations (Fig. 5**A**)

Once the optimal path is constructed, we use the Wilcoxon rank-sum test to find differentially expressed (DE) genes for each path, representing key driver genes for the developmental trajectories. Fig. 5 shows the expression of top-rank DE genes for the PSM path (TBX16) and Hindbrain path (SOX3), such that their respective expression levels vary along the path (as well as in adjacent regions). Warga et al. (2013) have found TBX16 regulates intermediate mesoderm cell fate. Other studies (Dee et al., 2008; Vriz et al., 1996) have found SOX3 regulates neural fates and is involved in central nervous system development.

Finally, we perturb the expression profiles of key genes in all cells at the starting timepoint by multiplying their expression values with different levels of coefficient $\{10^{-3}, \cdots, 10^{3}\}$ to mimic overexpressing and knocking-out, and let `scNODE` predict trajectories for the perturbed gene expression. The perturbations are expected to result in changes in cell fates. We classify predicted cells with a Random Forest (RF) classifier (Breiman, 2001) trained with all unperturbed cells. We find overexpressing TBX16 expression results in the increment of PSM cell ratios (Fig. 5**C**) to over 70%, while perturbing random non-DE genes leads to no changes in cell ratios. Moreover, the ratio of Hindbrain cells decreased by around 25% when we overexpressed SOX3 expressions (Supplementary Fig. S9). These results indicate that `scNODE` learns an interpretable latent space, where we can detect development-related DE genes and conduct *in silico* perturbation analysis.

4. Conclusion

We propose a generative model called `scNODE` to predict gene expression during cell development. Given a time-series scRNA-seq data set, our model can both interpolate and extrapolate gene expression. Predicting gene expression for unobserved timepoints enables critical downstream analyses, such as differential gene expression analysis, perturbation analysis, and trajectory inference. To make accurate predictions, `scNODE` uses a non-linear dimensionality reduction approach, neural ODE, and dynamic regularization. The regularization improves the robustness of the learned latent representation to distribution shifts in unobserved timepoints, resulting in better predictive performance.

We find that the primary challenge for the temporal scRNA-seq prediction task is extrapolating to multiple timepoints beyond the last observed timepoint, as the accuracy deteriorates the farther timepoint predictions. Moreover, time units in predictions are defined by time intervals in the data. For example, a generative model cannot accurately produce gene expression for every hour if the model is trained on observations that are only measured every day, especially when the development is rapid or contains complex trajectories.

In future work, we will incorporate knowledge about cell proliferation - which is known to be important to cellular differentiation - into the model to improve extrapolating

performance. Also, although RNA velocity methods can be used as complementary approaches to `scNODE` to learn fine-grained dynamics from coarse-grained timepoints. We will also incorporate other modalities, such as single-cell chromatin accessibility (scATAC) datasets, which exhibit a regulatory mechanism that scRNA-seq data might not capture.

References

T. Akiba, S. Sano, T. Yanase, T. Ohta, and M. Koyama. Optuna: A next-generation hyperparameter optimization framework. In *Proceedings of the 25th ACM SIGKDD international conference on knowledge discovery & data mining*, pages 2623–2631, 2019.

V. Bergen, M. Lange, S. Peidli, F. A. Wolf, and F. J. Theis. Generalizing RNA velocity to transient cell states through dynamical modeling. *Nature biotechnology*, 38 (12):1408–1414, 2020.

V. Bergen, R. A. Soldatov, P. V. Kharchenko, and F. J. Theis. RNA velocity—current challenges and future perspectives. *Molecular systems biology*, 17(8):e10282, 2021.

L. Breiman. Random forests. *Machine learning*, 45:5–32, 2001.

D. Calderon, R. Blecher-Gonen, X. Huang, S. Secchia, J. Kentro, R. M. Daza, B. Martin, A. Dulja, C. Schaub, C. Trapnell, et al. The continuum of Drosophila embryonic development at single-cell resolution. *Science*, 377(6606):eabn5800, 2022.

Y. Cao, P. Yang, and J. Y. H. Yang. A benchmark study of simulation methods for single-cell rna sequencing data. *Nature communications*, 12(1):6911, 2021.

G. Chen, B. Ning, and T. Shi. Single-cell RNA-seq technologies and related computational data analysis. *Frontiers in genetics*, page 317, 2019.

R. T. Chen, Y. Rubanova, J. Bettencourt, and D. K. Duvenaud. Neural ordinary differential equations. *Advances in neural information processing systems*, 31, 2018.

W. Chen, O. Guillaume-Gentil, P. Y. Rainer, C. G. Gäbelein, W. Saelens, V. Gardeux, A. Klaeger, R. Dainese, M. Zachara, T. Zambelli, et al. Live-seq enables temporal transcriptomic recording of single cells. *Nature*, 608(7924):733–740, 2022a.

Z. Chen, W. C. King, A. Hwang, M. Gerstein, and J. Zhang. DeepVelo: Single-cell transcriptomic deep velocity field learning with neural ordinary differential equations. *Science Advances*, 8(48):eabq3745, 2022b.

M. Connor, G. Canal, and C. Rozell. Variational autoencoder with learned latent structure. In *International conference on artificial intelligence and statistics*, pages 2359–2367. PMLR, 2021.

I. Csiszár. I-divergence geometry of probability distributions and minimization problems. *The annals of probability*, pages 146–158, 1975.

M. Cuturi. Sinkhorn distances: Lightspeed computation of optimal transport. *Advances in neural information processing systems*, 26, 2013.

C. T. Dee, C. S. Hirst, Y.-H. Shih, V. B. Tripathi, R. K. Patient, and P. J. Scotting. Sox3 regulates both neural fate and differentiation in the zebrafish ectoderm. *Developmental Biology*, 320(1):289–301, 2008.

J. Ding, A. Condon, and S. P. Shah. Interpretable dimensionality reduction of single cell transcriptome data with deep generative models. *Nature communications*, 9 (1):2002, 2018.

J. Ding, N. Sharon, and Z. Bar-Joseph. Temporal modelling using single-cell transcriptomics. *Nature Reviews Genetics*, 23(6):355–368, 2022.

J. A. Farrell, Y. Wang, S. J. Riesenfeld, K. Shekhar, A. Regev, and A. F. Schier. Single-cell reconstruction of developmental trajectories during zebrafish embryogenesis. *Science*, 360(6392):eaar3131, 2018.

J. S. Fleck, S. M. J. Jansen, D. Wollny, F. Zenk, M. Seimiya, A. Jain, R. Okamoto, M. Santel, Z. He, J. G. Camp, et al. Inferring and perturbing cell fate regulomes in human brain organoids. *Nature*, pages 1–8, 2022.

C. H. Grønbech, M. F. Vording, P. N. Timshel, C. K. Sønderby, T. H. Pers, and O. Winther. scVAE: variational auto-encoders for single-cell gene expression data. *Bioinformatics*, 36(16):4415–4422, 2020.

X. Han, Z. Zhou, L. Fei, H. Sun, R. Wang, Y. Chen, H. Chen, J. Wang, H. Tang, W. Ge, et al. Construction of a human cell landscape at single-cell level. *Nature*, 581 (7808):303–309, 2020.

J. S. Herman, n. Sagar, and D. Gruen. FateID infers cell fate bias in multipotent progenitors from single-cell RNA-seq data. *Nature methods*, 15(5):379–386, 2018.

G. Huguet, D. S. Magruder, A. Tong, O. Fasina, M. Kuchroo, G. Wolf, and S. Krishnaswamy. Manifold interpolating optimal-transport flows for trajectory inference. *Advances in Neural Information Processing Systems*, 35:29705–29718, 2022.

B. Hwang, J. H. Lee, and D. Bang. Single-cell RNA sequencing technologies and bioinformatics pipelines. *Experimental & molecular medicine*, 50(8):1–14, 2018.

M. Ipsen. Evolutionary reconstruction of networks. *Function and regulation of cellular systems*, pages 241–249, 2004.

P. Kidger. On neural differential equations. *arXiv preprint arXiv:2202.02435*, 2022.

D. P. Kingma, M. Welling, et al. An introduction to variational autoencoders. *Foundations and Trends® in Machine Learning*, 12(4):307–392, 2019.

D. Kobak and P. Berens. The art of using t-SNE for single-cell transcriptomics. *Nature communications*, 10(1):5416, 2019.

G. La Manno, R. Soldatov, A. Zeisel, E. Braun, H. Hochgerner, V. Petukhov, K. Lidschreiber, M. E. Kastriti, P. Lönnerberg, A. Furlan, et al. RNA velocity of single cells. *Nature*, 560(7719):494–498, 2018.

Q. Li. scTour: a deep learning architecture for robust inference and accurate prediction of cellular dynamics. *Genome Biology*, 24(1):1–33, 2023.

W. V. Li and J. J. Li. A statistical simulator scDesign for rational scrna-seq experimental design. *Bioinformatics*, 35(14):i41–i50, 2019.

E. Lin, S. Mukherjee, and S. Kannan. A deep adversarial variational autoencoder model for dimensionality reduction in single-cell RNA sequencing analysis. *BMC bioinformatics*, 21(1):1–11, 2020.

B. Liu, X. Hu, K. Feng, R. Gao, Z. Xue, S. Zhang, Y. Zhang, E. Corse, Y. Hu, W. Han, et al. Temporal single-cell tracing reveals clonal revival and expansion of precursor exhausted t cells during anti-PD-1 therapy in lung cancer. *Nature Cancer*, 3(1):108–121, 2022.

M. D. Luecken and F. J. Theis. Current best practices in single-cell RNA-seq analysis: a tutorial. *Molecular systems biology*, 15(6):e8746, 2019.

M. Marouf, P. Machart, V. Bansal, C. Kilian, D. S. Magruder, C. F. Krebs, and S. Bonn. Realistic in silico generation and augmentation of single-cell rna-seq data using generative adversarial networks. *Nature communications*, 11(1):166, 2020.

H. Matsumoto, H. Kiryu, C. Furusawa, M. S. Ko, S. B. Ko, N. Gouda, T. Hayashi, and I. Nikaido. SCODE: an efficient regulatory network inference algorithm from single-cell RNA-seq during differentiation. *Bioinformatics*, 33(15):2314–2321, 2017.

L. McInnes, J. Healy, and J. Melville. Umap: Uniform manifold approximation and projection for dimension reduction. *arXiv preprint arXiv:1802.03426*, 2018.

N. Moris, C. Pina, and A. M. Arias. Transition states and cell fate decisions in epigenetic landscapes. *Nature Reviews Genetics*, 17(11):693–703, 2016.

X. Qiu, S. Ding, and T. Shi. From understanding the development landscape of the canonical fate-switch pair to constructing a dynamic landscape for two-step neural differentiation. *PloS one*, 7(12):e49271, 2012.

X. Qiu, Q. Mao, Y. Tang, L. Wang, R. Chawla, H. A. Pliner, and C. Trapnell. Reversed graph embedding resolves complex single-cell trajectories. *Nature methods*, 14(10):979–982, 2017.

X. Qiu, Y. Zhang, J. D. Martin-Rufino, C. Weng, S. Hosseinzadeh, D. Yang, A. N. Pogson, M. Y. Hein, K. H. J. Min, L. Wang, et al. Mapping transcriptomic vector fields of single cells. *Cell*, 185(4):690–711, 2022.

D. Risso, F. Perraudeau, S. Gribkova, S. Dudoit, and J.-P. Vert. A general and flexible method for signal extraction from single-cell RNA-seq data. *Nature communications*, 9(1):284, 2018.

W. Saelens, R. Cannoodt, H. Todorov, and Y. Saeys. A comparison of single-cell trajectory inference methods. *Nature biotechnology*, 37(5):547–554, 2019.

G. Schiebinger, J. Shu, M. Tabaka, B. Cleary, V. Subramanian, A. Solomon, J. Gould, S. Liu, S. Lin, P. Berube, et al. Optimal-transport analysis of single-cell gene expression identifies developmental trajectories in reprogramming. *Cell*, 176(4):928–943, 2019.

T. Sun, D. Song, W. V. Li, and J. J. Li. scDesign2: a transparent simulator that generates high-fidelity single-cell gene expression count data with gene correlations captured. *Genome biology*, 22(1):163, 2021.

V. Svensson, A. Gayoso, N. Yosef, and L. Pachter. Interpretable factor models of single-cell RNA-seq via variational autoencoders. *Bioinformatics*, 36(11):3418–3421, 2020.

A. Tong, J. Huang, G. Wolf, D. Van Dijk, and S. Krishnaswamy. Trajectorynet: A dynamic optimal transport network for modeling cellular dynamics. In *International conference on machine learning*, pages 9526–9536. PMLR, 2020.

D. Tran, H. Nguyen, B. Tran, C. La Vecchia, H. N. Luu, and T. Nguyen. Fast and precise single-cell data analysis using a hierarchical autoencoder. *Nature communications*, 12(1):1029, 2021.

C. Trapnell, D. Cacchiarelli, J. Grimsby, P. Pokharel, S. Li, M. Morse, N. J. Lennon, K. J. Livak, T. S. Mikkelsen, and J. L. Rinn. The dynamics and regulators of cell fate decisions are revealed by pseudotemporal ordering of single cells. *Nature biotechnology*, 32(4):381–386, 2014.

S. Vriz, C. Joly, H. Boulekbache, and H. Condamine. Zygotic expression of the zebrafish Sox-19, an hmg box-containing gene, suggests an involvement in central nervous system development. *Molecular brain research*, 40(2):221–228, 1996.

D. Wang and J. Gu. VASC: dimension reduction and visualization of single-cell RNA-seq data by deep variational autoencoder. *Genomics, proteomics & bioinformatics*, 16(5):320–331, 2018.

P. Wang, C. Song, H. Zhang, Z. Wu, X.-J. Tian, and J. Xing. Epigenetic state network approach for describing cell phenotypic transitions. *Interface focus*, 4(3):20130068, 2014.

R. M. Warga, R. L. Mueller, R. K. Ho, and D. A. Kane. Zebrafish Tbx16 regulates intermediate mesoderm cell fate by attenuating Fgf activity. *Developmental biology*, 383(1):75–89, 2013.

A. Woicik, M. Zhang, J. Chan, J. Ma, and S. Wang. Extrapolating heterogeneous time-series gene expression data using Sagittarius. *Nature Machine Intelligence*, pages 1–15, 2023.

F. A. Wolf, F. K. Hamey, M. Plass, J. Solana, J. S. Dahlin, B. Göttgens, N. Rajewsky, L. Simon, and F. J. Theis. PAGA: graph abstraction reconciles clustering with trajectory inference through a topology preserving map of single cells. *Genome biology*, 20:1–9, 2019.

R. Xiang, W. Wang, L. Yang, S. Wang, C. Xu, and X. Chen. A comparison for dimensionality reduction methods of single-cell RNA-seq data. *Frontiers in genetics*, 12: 646936, 2021.

G. H. T. Yeo, S. D. Saksena, and D. K. Gifford. Generative modeling of single-cell time series with PRESCIENT enables prediction of cell trajectories with interventions. *Nature communications*, 12(1):3222, 2021.

L. Zappia, B. Phipson, and A. Oshlack. Splatter: simulation of single-cell RNA sequencing data. *Genome biology*, 18 (1):174, 2017.
