# Supplementary material for: scNODE : generative model for temporal single cell transcriptomic data prediction
Source: Bioinformatics. 2024 Sep 4;40(Suppl 2):ii146–54. doi: 10.1093/bioinformatics/btae393 (PMC11373355; doi:10.1093/bioinformatics/btae393)
Supplement: btae393_Supplementary_Data [file btae393_supplementary_data.zip › supplementary.pdf]

Supplementary Notes

S1. Related Works

One category of methods learns gene expression dynamics by inferring cell trajectories [Herman et al., 2018, Qiu et al., 2017, Saelens et al., 2019, Trapnell et al., 2014, Wolf et al., 2019]. However, most of them are based on pseudo-time inference, which estimates differential time for cells based on cell pair distances in low-dimensional spaces. Therefore, they cannot model developmental dynamics with respect to real physical time.

In addition, a category of approaches uses RNA velocity [Bergen et al., 2020, 2021, La Manno et al., 2018] to uncover developmental trends and underlying kinetics of gene expression. But they have inevitable limitations on gene expression prediction because they use linear ordinary differential equations (ODEs) [Matsumoto et al., 2017] that fail to capture complex gene expression dynamics, deeply sequenced datasets to obtain spliced and unspliced counts as prior information [Farrell et al., 2023], or have to use RNA velocity pre-computed from gene expression [Chen et al., 2022b] that causes double dipping [Ball et al., 2020] problems. Furthermore, most RNA velocity models are non-generative and infeasible for predicting gene expression.

Single-cell gene expression prediction has been investigated in previous studies [Cao et al., 2021]. For example, Marouf et al. [2020] proposes the use of generative adversarial networks (GAN) to generate realistic augmentations for specific cell types. Moreover, Li and Li [2019], Risso et al. [2018], Sun et al. [2021], Zappia et al. [2017] use statistical models to fit distributions of observed single-cell RNA sequencing (scRNA-seq) data and use the learned information to generate simulations. These models can make accurate predictions that reflect the properties of the experimental data. However, they are designed for gene expression measured at a single time snapshot and do not consider developmental dynamics. Therefore, they are infeasible for predicting temporal scRNA-seq data.

Recently, a few generative methods have introduced procedures to understand cell differentiation based on physical time and predict unobserved timepoints. Sagittarius method [Woicik et al., 2023] predicts gene expressions at future timepoints but mainly focuses on bulk RNA-seq data that requires cell matching between timepoints. This matching is hard to obtain for real-world scRNA-seq as the cells are lysed during the experiment. Although Live-seq [Chen et al., 2022a] enables temporal transcriptomic recording on the same cells, most existing single-cell datasets still have the cell lysing problem. In another line of works, Waddington-OT (WOT) [Schiebinger et al., 2019] interpolates between two timepoints by using an optimal transport (OT) framework to predict cell-cell couplings. However, it cannot extrapolate to future timepoints as the cell-cell coupling is unavailable when the subsequent timepoint is missing. TrajectoryNet [Tong et al., 2020] utilizes continuous normalizing flow to model the paths between cell populations of two time snapshots and predict interpolations. Therefore, WOT and TrajectoryNet are unable to extrapolate beyond the observed timepoints because they require gene expression measurements at two endpoints to learn intermediate cellular developments. In addition, Sha et al. [2024] jointly models developmental dynamics with cell growth. However, it focuses on reconstructing cellular trajectories and inferring underlying gene regulatory networks. It does not show abilities to predict extrapolation to timepoints beyond the measured time range.

PRESCIENT [Yeo et al., 2021] applies principal component analysis (PCA) to reduce high-dimensional gene space to a low-dimensional representation. It then applies neural ordinary differential equations (ODEs) [Chen et al., 2018] in this low-dimensional space to model the cell developmental trajectory. Multiple studies [Ding et al., 2018, Tran et al., 2021, Xiang et al., 2021] have found that PCA-based low-dimensional representations of scRNA-seq expression cannot capture complex relationships in the highly heterogeneous single-cell data. Thus, PCA may have the issue of overcrowding representation [Kobak and Berens, 2019, Tran et al., 2021], where cells of different types are poorly separated in PCA-based low-dimensional space, and cellular variations are lost. MIOFlow [Huguet et al., 2022] replaces PCA with a geodesic variational autoencoder (VAE) to learn non-linear low-dimensional representations that better retain cellular variations. However, this method fixes its low-dimensional space at the beginning, followed by neural ODE modeling. We hypothesize that a fixed low-dimensional representation obtained using observed timepoints may not generalize to an unobserved timepoint, if its underlying distribution differs substantially. Therefore, these methods either use less effective dimensionality reduction approaches or freeze low-dimensional representations so that they have inaccurate predictions at unmeasured timepoints.

On the contrary, our `scNODE` uses VAE, a non-linear dimensionality reduction method that captures complex cell relationships. `scNODE` also updates the low-dimensional latent space when learning developmental dynamics, such that the latent space considers overall developments and is more robust against

distribution shifts.

S2. Single-Cell Dataset and Pre-Processing

We use three publicly available single-cell RNA sequencing (scRNA-seq) datasets. The ZB dataset is downloaded from Single Cell Portal with identifier SCP126. The DR data is downloaded from https://shendure-web.gs.washington.edu/content/members/DEAP_website/public/ and SC data are available at https://broadinstitute.github.io/wot/tutorial/. For all datasets, we use data after removing batch effects among different timepoints.

For each task, we first select highly variable genes (HVGs). HVGs are a subset of genes that contribute strongly to cell-to-cell variation within a cell population. Using only HVGs for analysis helps remove data noise and reduce computational costs. In each case, we detect the top 2000 HVGs only from training timepoints to avoid data leakage. We use *Scanpy* [Wolf et al., 2018] and *Seurat* [Hao et al., 2021] to detect HVGs.

We then normalize expression to remove cell-specific bias before conducting experiments. Specifically, given the unique molecular identifier (UMI) count expression of cell i as $\mathbf{X}_i \in \mathbb{Z}^p_{\geq 0}$ where $\mathbb{Z}_{\geq 0} = \{0, 1, 2, \cdots\}$, we normalize it by total counts over all genes

$$\mathbf{X}_i = \frac{\mathbf{X}_i}{\sum_{j=1}^{p} \mathbf{X}_i} * 10^4, \tag{S1}$$

followed by the log-transformation

$$\mathbf{X}_{ij} = \log(\mathbf{X}_{ij} + 1). \tag{S2}$$

The normalization is cell-specific so that there is no data leakage between training and testing data. Because SC datasets only provide normalized expression data, we normalize the other two datasets.

Table S1: The leave-out timepoints (testing timepoints) for three defined tasks in each dataset. The timepoint index starts with 0. Since the SC dataset has more timepoints, we leave out more timepoints than other datasets in its hard task.

**Dataset**	**# Timepoints**	**Leave-Out Timepoints (Testing Timepoints)**		
		Easy Task	Medium Task	Hard Task
**ZB**	12	4, 6, 8	10, 11	2,4,6,8,10,11
**DR**	11	4, 6, 8	8, 9, 10	2,4,6,8,9,10
**SC**	19	5, 10, 15	16, 17, 18	5,7,9,11,15,16,17,18

S3. `scNODE` Training and Inference

Our `scNODE` is implemented with *Pytorch 1.13* [Paszke et al., 2019] and is trained end-to-end. `scNODE` training consists of two main steps. `scNODE` first pre-trains the VAE component (encoder Enc_ϕ and decoder Dec_θ) with all cells of training timepoints. We use Adam optimizer to pre-train `scNODE` with a learning rate of 0.001 and 200 iterations. Then, `scNODE` optimizes both VAE and neural ODE components by minimizing the $\mathcal{L}_{\text{dyn}}$. We adopt batch training and use Adam optimizer to train `scNODE` with a learning rate of 0.001 and 1000 iterations. At each training iteration, we randomly select 32 cells at $t = 0$ as a batch and predict for every training timepoint $t \in \mathcal{T}$. Because the Wasserstein distance computation is expensive, batch training improves training efficiency and enables `scNODE` usage on large-scale datasets. We use *geomloss* [Feydy et al., 2019] to compute Wasserstein distance with blur = 0.05 and scaling = 0.5. Pseudo-codes of `scNODE` are provided in Algorithm S1.

Specifically, `scNODE` training loss function (Eq. 7 in the main manuscript) contains a dynamic regularization term to pose cellular dynamics to the latent space and combines structure relation and temporal development of cells. This leads to an informative, interpretable, and robust latent space. At each training timepoint $t \in \mathcal{T}$, we use the encoder to compute latent variables $\mathbf{Z}^{(t)}_{\text{enc}}$ for $\mathbf{X}^{(t)}$ and then compute the Wasserstein distance between it and latent variables $\mathbf{Z}^{(t)}$ generated from neural ODE. Notice at the first timepoint $t = 0$, its latent variable $\mathbf{Z}^{(0)}$ is computed from VAE encoding, such that the regularization $\text{Wass}\left(\mathbf{Z}^{(0)}_{\text{enc}},\ \mathbf{Z}^{(0)}\right) \approx 0$. But for the latter training timepoints, this regularization enforces similarities between structure latent representations from VAE and the dynamic manifold from neural ODE.

At inference, `scNODE` first uses the trained encoder Enc_ϕ to map gene expression at the first timepoint to the latent space and then samples latent variables for the required number of cells from the latent Gaussian distribution. Then, `scNODE` uses the ODE solver to predict latent variables of these cells at required timepoints and map them back to the gene space through the decoder Dec_θ. Our `scNODE` can predict gene expression at any timepoints, including those observed and unobserved ones.

Algorithm S1 `scNODE`

Input: The set of measured timepoint indices $\mathcal{T}$; expression matrices $\{\mathbf{X}^{(t)} \mid t \in \mathcal{T}\}$; hyperparameters Δt, λ, and β; randomly initialized neural networks Enc_ϕ, Dec_θ, and Drift_ω.
(Step I: Pre-Training)
$\mathbf{X}_{\text{ALL}} = \text{CONCAT}\left(\mathbf{X}^{(t)} \mid t \in \mathcal{T}\right)$ // concatenate cells from training timepoints
Optimize ϕ and θ to minimize $\mathcal{L}_{\text{pre}}$ (Eq. 2)
(Step II: Dynamic Modelling)
Optimize ϕ, θ, and ω to minimize $\mathcal{L}_{\text{dyn}}$ (Eq. 7)
Output: ϕ, θ, and ω

S4. Baseline Models

We compare `scNODE` with the following baseline models.

- **PRESCIENT**: Yeo et al. [2021] proposes a generative model, called Potential eneRgy undErlying Single Cell gradIENTs (`PSCIENT`), to learn the differentiation landscape from single-cell time-series gene expression data. `PRESCIENT` maps gene expression to the PCA space and models cellular differentiation with neural ordinary differential equation (ODE). `PRESCIENT` suggests using cell proliferation information to improve prediction. But cell proliferation is not available in general cases. Therefore, we do not use such information in our experiments for all methods. We use Python codes on GitHub (https://github.com/gifford-lab/prescient) to run `PRESCIENT`.

- **MIOFlow**: Huguet et al. [2022] integrate geodesic VAE and neural ODE to model the paths of cells in a latent space while cellular structures are preserved. Specifically, `MIOFlow` first learns low-dimensional cell representation with a geodesic VAE such that latent space keeps the geometry of data manifold. `MIOFlow` then models cellular differentiation with neural ODE in this latent space. We use Python codes on GitHub (https://github.com/KrishnaswamyLab/MIOFlow) to run `MIOFlow`.

Waddington-OT [Schiebinger et al., 2019] and TrajectoryNet [Tong et al., 2020] are the other two generative methods. However, they can only predict interpolations between timepoints. Moreover, Yeo et al. [2021] and Huguet et al. [2022] have shown that PRESCIENT and MIOFlow perform better than these two methods. In this work, we focus on generative models that can both interpolate and extrapolate gene expressions, thus excluding Waddington-OT and TrajectoryNet in the comparison.

S5. Hyper-Parameter Tuning

For each method, we select hyperparameters that yield the minimum averaged Wasserstein distance using 3-fold cross-validation. For example, if the dataset has training timepoints $t = 0, 1, 2$ and we want to predict gene expression at testing timepoint $t = 3$. In cross-validation, we equally split the cells at every timepoint of $t = 0, 1, 2$ into three subsets, train the model on two of them, and validate it on the rest one. We repeat this process three times to ensure each subset is used as the validation set once and record the averaged Wasserstein distance on three validation sets. We use Optuna Akiba et al. [2019] to automatically determine the optimal hyperparameters and use sufficiently large hyperparameter ranges for search and evaluation. The search spaces of hyperparameters are shown in Table S2.

Specifically, we set the latent space dimension as 50 and step size $\Delta t = 0.1$ for our `scNODE` and all baseline methods. State-of-the-art methods [Heumos et al., 2023, Townes et al., 2019, Tran et al., 2021] also generally choose a latent space of tens of dimensions. For a fair comparison, we set the same latent space size for all methods. In all experiments, we set the Kulback-Leibler (KL) regularization coefficient $\lambda = 0$ in `scNODE` pertaining (Eq. 2 in the manuscript). Because we have tested various λ values and find $\lambda = 0$ leads to the best `scNODE` performance. We hypothesize that this is because cell latent representations do not necessarily follow the prior normal distribution $\mathcal{N}(0, 1)$ so that the KL penalty

may introduce biases in the latent representation learning. Moreover, step size $\Delta t = 0.1$ implies the neural ODE interpolates 10 uniformly spaced timepoints between two consecutive training timepoints. A small step size increases dynamic resolution, and a larger step size reduces computational costs. Previous works [Huguet et al., 2022, Yeo et al., 2021] also use $\Delta t = 0.1$ as a proper trade-off between efficiency and accuracy. We train each method for sufficient iterations to ensure they converge. Specifically, we train `scNODE` 200 pre-training iterations and 1000 training iterations, `PRESCIENT` 500 epochs, `MIOFlow` 1000 pre-training iterations and 100 training epochs. We let every model predict 2000 cells at each predicted timepoint to ensure a fair metric comparison. The datasets used in our experiments have thousands of cells at each timepoint, so we believe 2000 is a reasonable number of predicted cells.

Table S2: Hyperparameters search space of `scNODE` and baseline methods. "None" means no hidden layers.

**Model**	**Hyperparameters Search Space**
`scNODE`	$d = 50$, $\lambda = 0$<br>regularize coefficient $\beta \in [0.0, 10.0]$<br>encoder network size $\in \{\text{None}, [d], [d, d]\}$<br>decoder network size $\in \{\text{None}, [d], [d, d]\}$<br>drift network size $\in \{\text{None}, [d], [d, d]\}$
`PRESCIENT`	$d = 50$<br># hidden layers $\in \{1, 2, 3\}$<br>sd $\in [0.0, 1.0]$<br>tau $\in [0.0, 0.1]$<br>gradient clip $\in [0.0, 1, 0]$
`MIOFlow`	$d = 50$<br>gae embedded dim $\in \{10, 50, 100\}$<br>part encoder layers $\in \{[2000, 100], [2000, 100, 100]\}$<br>layers $\in \{[16], [16, 16], [16, 32, 16]\}$<br>$\lambda \in [1.0, 100.0]$

S6. More on experiments

S6.1. scNODE can accurately predict expression at unmeasured timepoints

Table S3: Wasserstein distance of predictions in the easy (interpolation only) and medium (extrapolation only) tasks for all three real-world datasets. We report average(std.) Wasserstein distance over five trials. Bold numbers denote the best prediction, and underlined numbers represent the second best.

**Dataset**	**ZB**				
	**Easy (interpolation only)**			**Medium (extrapolation only)**	
	$t = 4$	$t = 6$	$t = 8$	$t = 10$	$t = 11$
`scNODE`	**433.06(7.1)**	**393.08(3.9)**	**474.37(3.5)**	**608.79(5.1)**	**684.15(11.3)**
`MIOFlow`	<u>458.60(13.6)</u>	<u>406.51(6.2)</u>	<u>487.62(5.5)</u>	<u>620.52(8.8)</u>	<u>712.38(25.8)</u>
`PRESCIENT`	840.30(0.0)	693.66(0.0)	705.99(0.0)	989.87(0.0)	1045.14(0.0)

**Dataset**	**DR**					
	**Easy (interpolation only)**			**Medium (extrapolation only)**		
	$t = 4$	$t = 6$	$t = 8$	$t = 8$	$t = 9$	$t = 10$
`scNODE`	<u>348.18(1.4)</u>	**404.47(1.6)**	**490.55(2.2)**	**546.81(9.0)**	<u>575.92(24.6)</u>	<u>717.09(27.9)</u>
`MIOFlow`	**346.71(1.0)**	<u>406.16(2.2)</u>	<u>497.42(2.5)</u>	<u>553.26(2.5)</u>	594.77(19.2)	775.86(38.0)
`PRESCIENT`	449.68(0.0)	476.85(0.0)	519.6(0.0)	600.77(0.0)	**553.21(0.0)**	**709.49(0.0)**

We compare `scNODE` 's performance with baseline methods for predicting gene expression at left-out timepoints. Tables S3 and S4 show the Wasserstein distance of left-out testing timepoints for defined easy, medium, and hard tasks. We report Wasserstein distances averaged on five repeating trials. `scNODE` clearly outperforms the baselines in most cases. In other cases where `scNODE` has the second best predictions,

Dataset	SC					
	Easy (interpolation only)			Medium (extrapolation only)		
	$t = 5$	$t = 10$	$t = 15$	$t = 16$	$t = 17$	$t = 18$
scNODE	58.97(1.7)	**133.91(3.7)**	**118.50(3.2)**	**130.26(6.5)**	**123.80(1.9)**	**128.08(2.5)**
MIOFlow	**56.49(1.8)**	136.15(1.85)	122.56(2.3)	154.48(7.0)	154.52(5.3)	172.74(8.2)
PRESCIENT	104.99(0.0)	138.45(0.0)	154.50(0.0)	177.17(0.0)	163.12(0.0)	168.07(0.0)

scNODE has a similar performance as MIOFlow and significantly performs better than PRESCIENT. Moreover, in medium and hard tasks, where extrapolations are required, scNODE can have significant superiority over baselines. For example, at $t = 15$ of SC hard task (in Table S4), Wasserstein distance of scNODE predictions is around 132, while that of PRESCIENT and MIOFlow are about 162 and 150 respectively. Therefore, scNODE has better overall performance, especially in more challenging tasks.

Table S4: Wasserstein distance of predictions in hard tasks (inter- and extrapolation). We report average(std.) Wasserstein distance over five trials. **Bold** numbers denote the best prediction, and underlined numbers represent the second best.

Method	ZB / Hard Task					
	Interpolation				Extrapolation	
	$t = 2$	$t = 4$	$t = 6$	$t = 8$	$t = 10$	$t = 11$
scNODE	**579.10(2.2)**	**508.55(3.6)**	**440.92(4.7)**	**517.81(2.2)**	**652.36(3.1)**	**707.10(4.9)**
MIOFlow	580.18(7.5)	516.59(7.9)	453.61(3.4)	536.35(2.3)	671.23(15.9)	734.42(21.7)
PRESCIENT	1381.96(0.0)	1002.62(0.0)	730.974(0.0)	701.29(0.0)	916.51(0.0)	973.17(0.0)

Method	DR / Hard Task					
	Interpolation			Extrapolation		
	$t = 2$	$t = 4$	$t = 6$	$t = 8$	$t = 9$	$t = 10$
scNODE	445.82(4.7)	**464.78(2.6)**	535.78(3.8)	**600.18(7.0)**	585.60(6.8)	**718.20(3.2)**
MIOFlow	**443.56(6.5)**	469.51(1.7)	**532.93(2.5)**	617.48(9.8)	680.41(18.6)	852.02(37.2)
PRESCIENT	524.38(0.0)	511.61(0.0)	539.38(0.0)	621.31(0.0)	**575.45(0.0)**	718.56(0.0)

Method	SC / Hard Task							
	Interpolation				Extrapolation			
	$t = 5$	$t = 7$	$t = 9$	$t = 11$	$t = 15$	$t = 16$	$t = 17$	$t = 18$
scNODE	55.22(0.9)	**59.89(1.0)**	**103.26(2.0)**	**140.81(2.4)**	**132.86(7.3)**	**148.89(10.8)**	**137.90(10.6)**	**151.13(12.7)**
MIOFlow	**55.07(2.0)**	61.80(1.6)	108.72(2.1)	156.51(6.9)	162.12(19.1)	191.40(30.2)	189.39(35.3)	215.74(49.9)
PRESCIENT	85.36(0.0)	87.47(0.0)	114.16(0.0)	142.03(0.0)	150.53(0.0)	161.59(0.0)	147.23(0.0)	155.06(0.0)

We also visualize ground truth and model predictions in the 2D UMAP space to qualitatively compare scNODE with baseline models. Specifically, in each case, we first map all timepoints of ground truth to the 50-dimensional PCA space and then compute the 2D UMAP embedding of them. Then we project each model's predictions to this UMAP space with the PCA and UMAP transformation initialized from ground truth. We use the *umap* package (https://github.com/lmcinnes/umap) and set n_neighbors=50 and min_dist=0.1 when computing UMAP embeddings. Fig. S1, S2, and S3 show that our scNODE predictions better align with ground truth.

Unsurprisingly, extrapolating into the future is more difficult than interpolating because the training data may not contain sufficient information about future developments. Moreover, the distribution of gene expression at future timepoints may shift drastically from training data, so extrapolation is challenging for model generalization. Therefore, to evaluate model predictions in extrapolating multiple timepoints and test the generalization and stability of generative models against distribution shift, we let each model predict different future timepoints and track its performance. Specifically, in the ZB, DR, and SC datasets, we remove the last $\{1, 2 \cdots, 5\}$ timepoints, train each model on the rest of the timepoints, and let them predict extrapolations. In each case, we re-do the preprocessing and re-tune all hyperparameters. Fig. S4 shows the averaged Wasserstein distance in each setting. We notice for all methods, their extrapolations become less accurate the farther out predictions are made. But scNODE consistently performs better in most cases. Especially when extrapolating five timepoints, scNODE significantly outperforms the other

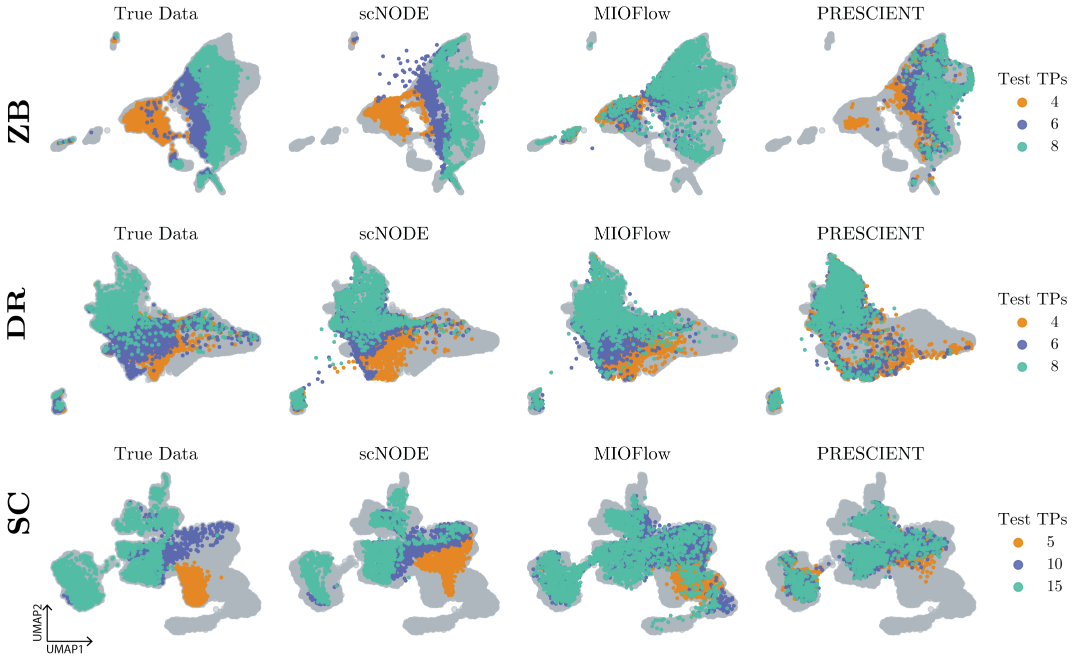

Fig. S1: UMAP visualizations of true and model predictions in easy tasks. Gray points represent training data.

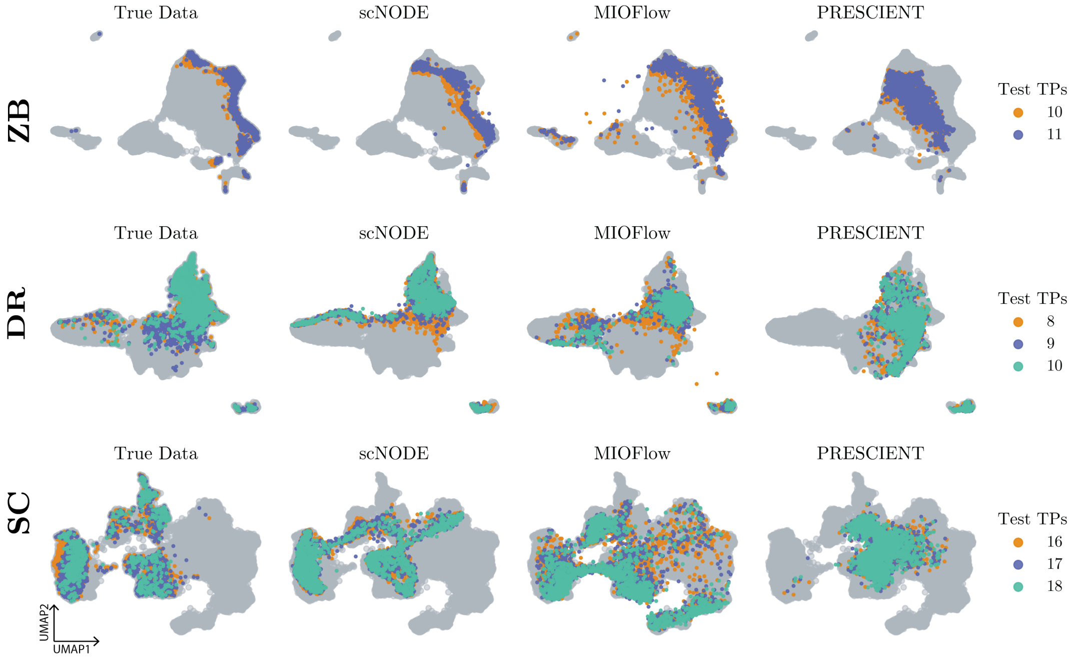

Fig. S2: UMAP visualizations of true and model predictions in medium tasks. Gray points represent training data.

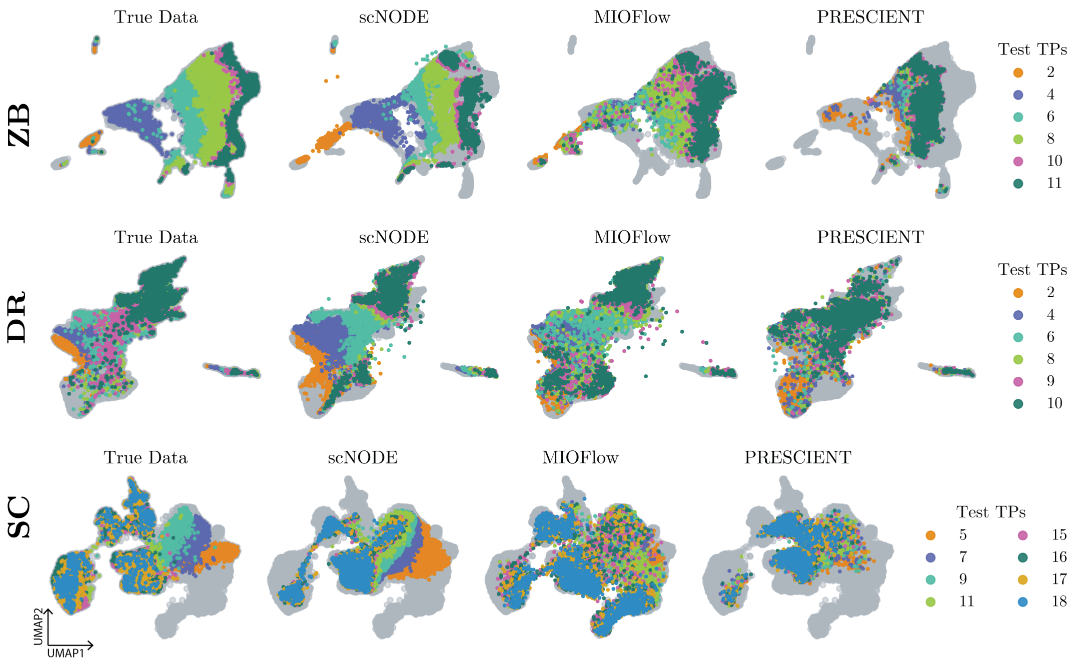

Fig. S3: UMAP visualizations of true and model predictions in hard tasks. Gray points represent training data.

two methods in DR and SC datasets, which implies that `scNODE` better generalizes to unobserved future timepoints that have shifted distributions. Therefore, although accurate extrapolation is a challenge [Woicik et al., 2023] in single-cell gene expression prediction, `scNODE` still demonstrates significant improvement over state-of-the-art methods in accurately extrapolating future timepoints.

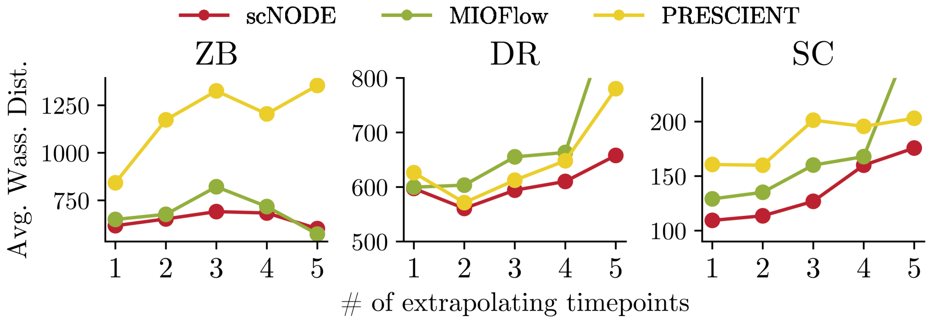

Fig. S4: Compare `scNODE` and baseline model predictions on extrapolating multiple timepoints in ZB, DR, and SC datasets. We show the Wasserstein distance between true and predicted expression averaged across all testing timepoints.

S6.2. `scNODE` *is more robust to distribution shift between observed and unobserved timepoints*

We show that `scNODE` is more robust to distribution shift and obtains significant improvements over baseline models when testing timepoints have substantially different distributions as training data. In three hard tasks, we compute the distribution shift level for each testing timepoint as the averaged pairwise ℓ_2 distance between cells from training and testing timepoints. Specifically, given training data matrix $\mathbf{X} \in \mathbb{R}^{n \times p}$ and testing data matrix $\mathbf{Y} \in \mathbb{R}^{m \times p}$. The distribution shift level is computed through

$$\text{distribution shift} = \frac{1}{mn} \sum_{i=1}^{n} \sum_{j=1}^{m} \| X_i - Y_j \|_2 \,. \tag{S3}$$

Hence, a higher value indicates the testing point has more significant differences from the training data. We choose to use pairwise ℓ_2 distance because it measures every possible cell pair between training and testing data, such that it is a comprehensive evaluation of distribution differences.

Then, we define scNODE 's improvement as the difference between its performance (calculated as Wasserstein distance between model predictions and ground truth) and the performance of the best baseline model

$$\texttt{scNODE}\text{ improvement at timepoint } t = \text{Wass}(\mathbf{X}^{(t)}, \widehat{X}^{(t)}_{\text{base}}) - \text{Wass}(\mathbf{X}^{(t)}, \widehat{X}^{(t)}_{\texttt{scNODE}}). \tag{S4}$$

Here, $\widehat{X}^{(t)}_{\text{base}}$ and $\widehat{X}^{(t)}_{\texttt{scNODE}}$ are predictions of the best baseline model and scNODE correspondingly. Since a lower Wasserstein distance indicates better predictions, a positive scNODE improvement value indicates scNODE outperforms baselines. Also, a higher value indicates that scNODE predictions achieve more improvements over the baseline.

We compute the Spearman's ρ correlation between distribution level and scNODE improvements for each testing timepoint. Before computing correlation, we remove outliers by fitting the data points with RANdom SAmple Consensus (RANSAC) regression, an outlier-robust regression algorithm, and remove the sample with the largest residual. Fig. 3 in the manuscript implies that scNODE improvements positively correlate with the distribution shift level. The correlations are $\rho = 0.5$, $\rho = 0.3$, and $\rho = 0.93$ for ZB, DR, and SC datasets, respectively. Therefore, scNODE is robust to distribution shift and shows more superiority when testing data are more different.

Last, we validate that scNODE learns a more robust latent space. We use the setting of leaving out the last three timepoints in all three datasets, where testing timepoints are located outside the training time range and have different distributions. We train all methods with training timepoints and compare the latent variables of testing timepoints generated by each model. Fig. S5 shows that PRESCIENT generates an over-mixing distribution of testing timepoints in the latent space, implying it fails at generating samples out of training distribution and has the worst overall performance. scNODE and MIOFlow can model temporal dynamics in the latent space and distinguish between different timepoints. However, MIOFlow latent variables are scattered and random, while scNODE has a more structured latent space, leading to accurate predictions at testing timepoints. This should be one reason for scNODE 's robustness against distribution shift.

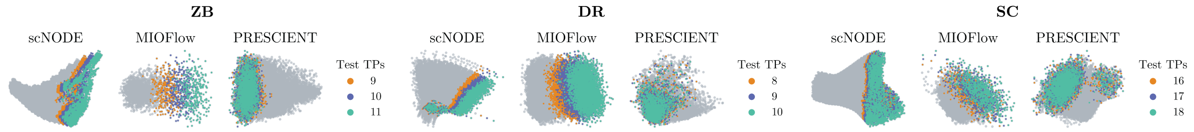

Fig. S5: Compare two-dimensional PCA visualization of latent variables when extrapolating the last three timepoints in ZB, DR, and SC datasets.

S6.3. Investigation of scNODE *hyperparameters*

We evaluate scNODE performance using different hyperparameter settings and give heuristic guidance on how to set hyperparameters in real-world scenarios. All experiments are conducted on the hard tasks. First, we vary the latent dimension from $\{25, 50, 75, \cdots, 200\}$. Table S5 indicates that scNODE predictions are robust to the size of the latent dimensionality. Users can choose to set a reasonable latent dimension based on a trade-off between accuracy and computational costs. State-of-the-art methods [Heumos et al., 2023, Townes et al., 2019, Tran et al., 2021] generally choose a latent space of 10 to 50 dimensions. We set the latent space size to 50 for all methods in our main comparison.

Then, we test the effects of the pre-training phase. Specifically, we compare the accuracy of scNODE predictions when excluding the pre-training step and using different numbers of training timepoints in pre-training. We compare two strategies of choosing pre-training timepoints: randomly select from training timepoints (denoted as "random") or pick the first several training timepoints (denoted as "first"). In each case, we run scNODE 5 trials. Fig. S6 shows that pre-training clearly improves predictions in all cases. Moreover, scNODE can outperform baselines even when not using all training timepoints in the pre-training step. This is because scNODE uses dynamic regularization to update the latent space. To validate the effects of latent space adjustment in scNODE , we also compare the predictions of scNODE when including and excluding the update of latent space. Excluding the adjustment of latent space means fixing the pre-trained VAE and only optimizing neural ODE parameters ω during model learning. Fig. S7 shows that latent space adjustment improves prediction accuracy. Overall, the ablation study shows that pre-training and latent space updates are necessary for accurate predictions.

scNODE uses a dynamic regularizer with hyperparameter β to update the VAE space dynamically such that it captures both cellular variations and the developmental dynamics of the scRNA-seq data. We test scNODE predictions when using different $\beta \in \{0.0, 0.25, 0.5, \cdots, 10.0\}$. Table S6 shows that the dynamic

Table S5: Performance of scNODE predictions on all datasets when using different latent space sizes (d).

**Dataset/Task**	**Latent Size (d)**									
	**25**	**50**	**75**	**100**	**125**	**150**	**175**	**200**	**avg.**	**std.**
**ZB/easy**	449.14	429.71	428.37	427.10	429.33	429.09	426.25	419.72	429.84	7.89
**ZB/medium**	648.85	661.06	634.73	636.39	623.58	622.91	630.47	633.52	636.44	12.02
**ZB/hard**	577.88	566.15	564.11	569.51	566.54	566.06	573.11	564.70	568.51	4.46
**DR/easy**	412.52	413.95	417.63	411.83	419.23	413.12	416.49	415.83	415.08	2.46
**DR/medium**	584.51	613.93	592.91	615.29	632.27	665.69	615.98	596.47	614.64	24.06
**DR/hard**	557.53	553.08	557.58	555.05	555.97	553.73	556.84	567.12	557.11	4.09
**SC/easy**	106.45	104.78	109.69	107.83	104.69	109.15	104.36	112.92	107.48	2.81
**SC/medium**	127.74	124.46	126.13	123.97	123.01	129.53	120.69	121.74	124.66	2.80
**SC/hard**	124.53	116.06	110.92	113.96	108.16	110.45	107.39	116.93	113.55	5.27

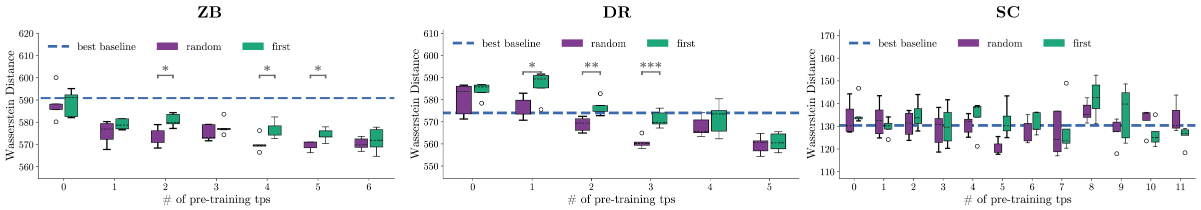

Fig. S6: Compare Wasserstein distances of scNODE predictions in hard tasks when using different numbers of pre-training timepoints. Asterisks indicate p-values of the t-test between two strategies. *: p-value≤ 0.05, **: p-value≤ 0.01, and ***: p-value≤ 0.001. The dotted lines indicate the best baseline performance.

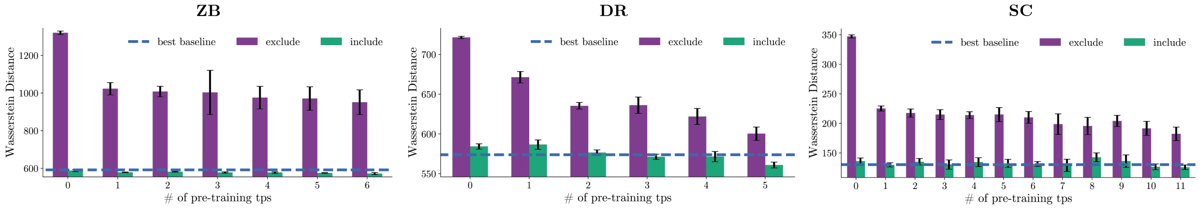

Fig. S7: Compare Wasserstein distances of scNODE predictions in hard tasks when including and excluding the update of latent space. Dotted lines indicate the best baseline performance.

regularization indeed improves model performance, as the best predictions are obtained with $\beta \neq 0$ (i.e., adopting the dynamic regularization) in all cases. Especially on the DR dataset, excluding the dynamic regularization significantly worsens `scNODE` predictions. The value of β affects model prediction accuracy, but `scNODE` is robust relative to the value of β. Moreover, `scNODE` performs better than baseline models in all tasks with a proper β value. In experiments for the main text, we tune β values for `scNODE` in each case.

Table S6: Wasserstein distance of `scNODE` predictions when varying the regularization coefficient β. We report Wasserstein distance averaged over testing timepoints. Bold numbers denote the best `scNODE` performance in each task.

Dataset/Task	Regularization Coefficient (β)									Best Baseline
	**0.0 (no regularizer)**	**0.25**	**0.5**	**0.75**	**1.0**	**2.5**	**5.0**	**7.5**	**10.0**	
**ZB/easy**	429.26	**427.89**	430.06	429.20	429.29	429.26	434.90	435.03	432.22	450.91
**ZB/medium**	645.88	646.16	658.26	658.09	647.16	647.74	647.35	**631.54**	647.58	666.45
**ZB/hard**	578.06	575.26	574.11	**570.97**	574.48	572.15	574.85	574.15	577.47	590.89
**DR/easy**	417.15	414.97	417.22	416.29	413.93	**412.45**	414.98	419.66	417.60	416.76
**DR/medium**	719.56	610.37	666.58	607.84	639.52	594.18	615.00	595.27	**589.59**	621.15
**DR/hard**	615.67	555.80	**552.68**	556.19	555.26	564.13	559.98	566.15	574.02	567.97
**SC/easy**	102.26	103.77	**102.13**	109.03	109.51	104.18	105.33	109.03	108.16	157.6
**SC/medium**	125.40	127.15	127.96	125.90	122.71	124.67	**122.32**	130.49	132.89	160.58
**SC/hard**	114.68	114.30	**112.53**	117.06	132.29	117.44	118.38	124.21	122.57	130.42

S6.4. `scNODE` *predictions help recover cell trajectories*

We validate that `scNODE` can aid cell trajectory inference using the hard task of all three datasets. Specifically, we leave out several timepoints (see hard tasks in Table S1) and let `scNODE` and baseline model predict them back, in order to see whether model predictions can help with reconstructing cell developmental trajectories. We apply partition-based graph abstraction (PAGA) [Wolf et al., 2019] to construct cell trajectories. PAGA is a method that computes the topological structure of cell populations and represents population structures in interpretable graphs. Specifically, we use Louvain clustering [Blondel et al., 2008] to first cluster cells in the 2D UMAP space and apply PAGA to construct a structural graph of these clusters. We use *Scanpy* for Louvain clustering.

In addition, we use the Ipsen-Mikhailov (IM) distance [Ipsen, 2004] to quantitatively measure the similarity between the cell trajectory graphs constructed in each case. $\mathrm{IM}(\mathcal{G}_1, \mathcal{G}_2)$ is a graph similarity measurement defined as the square-root difference between the Laplacian spectrum of graphs $\mathcal{G}_1$ and $\mathcal{G}_2$. It ranges from 0 to 1, where 0 indicates maximum similarity between two graph structures and 1 indicates maximum dissimilarity. We use *nedtrd* package [McCabe et al., 2020] to compute the IM distance. Fig. S8 shows that `scNODE` predictions help recovering cell trajectories as $\mathrm{IM}(\mathcal{G}_{\text{true}}, \mathcal{G}_{\text{scNODE}}) < \mathrm{IM}(\mathcal{G}_{\text{true}}, \mathcal{G}_{\text{removal}})$. Moreover, $\mathrm{IM}(\mathcal{G}_{\text{true}}, \mathcal{G}_{\text{scNODE}})$ being smaller than $\mathrm{IM}(\mathcal{G}_{\text{true}}, \mathcal{G}_{\text{MIOFlow}})$ and $\mathrm{IM}(\mathcal{G}_{\text{true}}, \mathcal{G}_{\text{PRESCIENT}})$ in all cases implies that `scNODE` predictions for missing timepoints best help to infer cell trajectories.

S6.5. `scNODE` *in perturbation analysis*

We use ZB data to test `scNODE` 's ability to help *in silico* perturbation analysis. We train `scNODE` with all timepoints of ZB data and construct the cellular state path in the latent space. The path is constructed with least action path (LAP), which has been used in previous works [Qiu et al., 2012, 2022, Wang et al., 2014] to construct cell fate transitions. The LAP method aims at finding the optimal path between two cell states while minimizing its action and transition time. Specifically, given starting point $\mathbf{X}_0$ and end point $\mathbf{X}_K$, LAP fins a path discretized as a sequence of K points $\mathcal{P} = \{\mathbf{X}_0, \cdots, \mathbf{X}_K\}$. For each segment constrained between $\mathbf{X}_{k-1}$ and $\mathbf{X}_k$, its tangential velocity is defined as $\mathbf{V}_k = \frac{(\mathbf{X}_k - \mathbf{X}_{k-1})}{\Delta}$ where Δ is the timestep taken by cells from $\mathbf{X}_{k-1}$. Therefore, we define the action $\mathcal{S}$ along the path $\mathcal{P}$ as

$$\mathcal{S} = \frac{1}{2} \sum_{k=1}^{K} \Big(\mathbf{V}_k - \mathrm{Drift}_\omega(\widetilde{\mathbf{X}}_k) \Big)^2 \Delta \quad \text{with } \widetilde{\mathbf{X}}_k = \frac{\mathbf{X}_{k-1} + \mathbf{X} - k}{2}. \tag{S5}$$

Here, LAP method aims to align the tangential velocity $\mathbf{V}_k$ with the differential velocity $\mathrm{Drift}_\omega(\widetilde{\mathbf{X}}_k)$ learned by `scNODE` , while having the least transition time. Therefore, the optimal path is

$$\widehat{\mathcal{P}} \;=\; \underset{\mathcal{P},\Delta}{\mathrm{argmin}}\, \mathcal{S} \;=\; \underset{\mathcal{P},\Delta}{\mathrm{argmin}}\, \frac{1}{2} \sum_{k=1}^{K} \Big(\mathbf{V}_k - \mathrm{Drift}_\omega(\widetilde{\mathbf{X}}_k) \Big)^2 \Delta. \tag{S6}$$

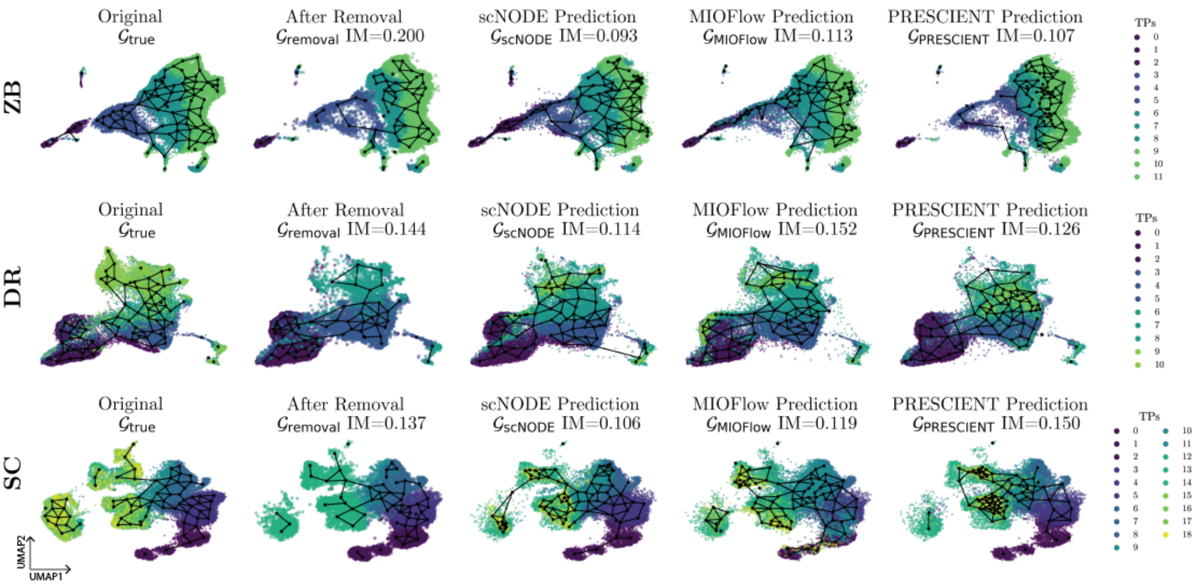

Fig. S8: `scNODE` helps infer cell trajectories. Here are cell trajectories of data with all timepoints, after the removal of timepoints in the hard task, and with model predictions. The connective structure is constructed with PAGA, where black nodes represent cell clusters and edges connect two nodes if their expressions are similar. We show the IM index between $\mathcal{G}_{\text{true}}$ and the corresponding graph.

Solving Eq. S6 consists of two iterative steps

(1) Minimize action by fixing path $\mathcal{P}$ and varying the timestep Δ through

$$\widehat{\Delta} = \underset{\Delta}{\operatorname{argmin}} \frac{1}{2} \sum_{k=1}^{K} \left(\frac{\mathbf{X}_k - \mathbf{X}_{k-1}}{\Delta} - \text{Drift}_{\omega}(\widetilde{\mathbf{X}}_k) \right)^2 \Delta. \tag{S7}$$

(2) Minimize action by fixing timestep $\widehat{\Delta}$ and varying path $\mathcal{P}$

$$\widehat{\mathcal{P}} = \underset{\mathbf{X}_1, \cdots, \mathbf{X}_{K-1}}{\operatorname{argmin}} \frac{1}{2} \sum_{k=1}^{K} \left(\frac{\mathbf{X}_k - \mathbf{X}_{k-1}}{\widehat{\Delta}} - \text{Drift}_{\omega}(\widetilde{\mathbf{X}}_k) \right)^2 \widehat{\Delta}. \tag{S8}$$

The starting ($\mathbf{X}_0$) and end point ($\mathbf{X}_K$) are fixed in the optimization.

We use *scipy.optimize.minimize* to solve these two objective functions. In our experiments, we construct two paths between the first timepoint and two cell populations (PSM and Hindbrain) with $K = 8$ (Fig. S9A). We set the starting point as the center of cells at the first timepoint (t=0) and the endpoint as the center of the cell population. We initialize timestep $\Delta = 1$ and $\mathcal{P}$ as equally spaced points from the starting to end points.

When finding the differentially expressed (DE) genes, we augment the LAP path with its nearest neighbors in the latent space (Fig. S9). Specifically, assuming $\mathcal{P} = \{\mathbf{X}_0, \cdots, \mathbf{X}_K\}$ is the LAP path from $\mathbf{X}_0$ to $\mathbf{X}_K$, we have only 8 cells on the path which is insufficient for DE detection. Therefore, for each $\mathbf{X}_k \in \mathcal{P}$, we find its nearest neighbors in the latent space in order to augment the path. We use *sklearn.neighbors.NearestNeighbors* to search for 10 nearest neighbors. The number of nearest neighbors affects DE detection, where fewer neighbors may be insufficient for good DE detection, and more neighbors may introduce noise. We select 10 neighbors for a proper trade-off between efficiency and accuracy. Then we can use *Scanpy* to detect DE genes for the augmented path with the Wilcoxon rank-sum test.

Lastly, we perturb the expression profile of key genes in all cells at the starting timepoint by multiplying their expression values with different levels of coefficient $\{10^{-3}, \cdots, 10^3\}$ to mimic overexpressing and knocking-out, and let `scNODE` predict trajectories for the perturbed gene expression. The perturbations are expected to result in changes in cell fates. Specifically, we overexpress TBX16 (DE gene of PSM) or SOX3 (DE gene of Hindbrain). We classify predicted cells with a Random Forest (RF) classifier trained with all unperturbed cells. We use *sklearn.ensemble.RandomForestClassifier* with default parameters as the classifier and train it with all unperturbed cell expression as well as cell type labels in original datasets. We compare cell ratios when perturbing DE gene and random non-DE gene and find overexpressing

TBX16 expression results in the increment of PSM cell ratios (Fig. S9**B**) to over 70%. Moreover, the ratio of Hindbrain cells decreased when overexpress SOX3 expressions (Fig. S9**C**). These indicate that detected DE genes are related to developmental dynamics. In applications of `scNODE` , these perturbations can involve multiple genes, use different cell populations, or be carried out at different timepoints.

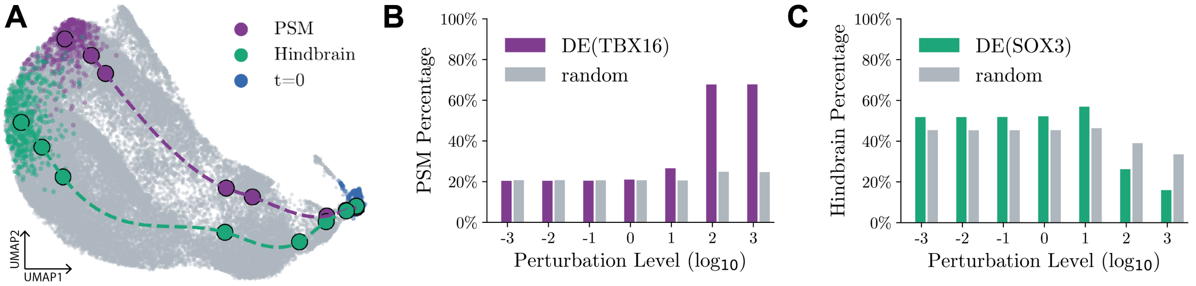

Fig. S9: `scNODE` perturbation analysis results. (**A**) 2D UMAP visualization of the least action path between cells at the starting point ($t = 0$) and PSM/Hindbrain cell populations. (**B**) The ratio of PSM cells in predictions of different perturbation levels when perturbing TBX16 or ten random non-DE genes. (**C**) The ratio of Hindbrain cells in predictions of different perturbation levels when perturbing SOX3 or ten random non-DE genes.

References

T. Akiba, S. Sano, T. Yanase, T. Ohta, and M. Koyama. Optuna: A next-generation hyperparameter optimization framework. In *Proceedings of the 25th ACM SIGKDD international conference on knowledge discovery & data mining*, pages 2623–2631, 2019.

T. M. Ball, L. M. Squeglia, S. F. Tapert, and M. P. Paulus. Double dipping in machine learning: problems and solutions. *Biological psychiatry. Cognitive neuroscience and neuroimaging*, 5(3):261, 2020.

V. Bergen, M. Lange, S. Peidli, F. A. Wolf, and F. J. Theis. Generalizing RNA velocity to transient cell states through dynamical modeling. *Nature biotechnology*, 38(12):1408–1414, 2020.

V. Bergen, R. A. Soldatov, P. V. Kharchenko, and F. J. Theis. RNA velocity—current challenges and future perspectives. *Molecular systems biology*, 17(8):e10282, 2021.

V. D. Blondel, J.-L. Guillaume, R. Lambiotte, and E. Lefebvre. Fast unfolding of communities in large networks. *Journal of statistical mechanics: theory and experiment*, 2008(10):P10008, 2008.

Y. Cao, P. Yang, and J. Y. H. Yang. A benchmark study of simulation methods for single-cell rna sequencing data. *Nature communications*, 12(1):6911, 2021.

R. T. Chen, Y. Rubanova, J. Bettencourt, and D. K. Duvenaud. Neural ordinary differential equations. *Advances in neural information processing systems*, 31, 2018.

W. Chen, O. Guillaume-Gentil, P. Y. Rainer, C. G. Gäbelein, W. Saelens, V. Gardeux, A. Klaeger, R. Dainese, M. Zachara, T. Zambelli, et al. Live-seq enables temporal transcriptomic recording of single cells. *Nature*, 608(7924):733–740, 2022a.

Z. Chen, W. C. King, A. Hwang, M. Gerstein, and J. Zhang. DeepVelo: Single-cell transcriptomic deep velocity field learning with neural ordinary differential equations. *Science Advances*, 8(48):eabq3745, 2022b.

J. Ding, A. Condon, and S. P. Shah. Interpretable dimensionality reduction of single cell transcriptome data with deep generative models. *Nature communications*, 9(1):2002, 2018.

S. Farrell, M. Mani, and S. Goyal. Inferring single-cell transcriptomic dynamics with structured latent gene expression dynamics. *Cell Reports Methods*, 3(9), 2023.

J. Feydy, T. Séjourné, F.-X. Vialard, S.-i. Amari, A. Trouvé, and G. Peyré. Interpolating between optimal transport and mmd using sinkhorn divergences. In *The 22nd International Conference on Artificial Intelligence and Statistics*, pages 2681–2690. PMLR, 2019.

Y. Hao, S. Hao, E. Andersen-Nissen, W. M. Mauck, S. Zheng, A. Butler, M. J. Lee, A. J. Wilk, C. Darby, M. Zager, et al. Integrated analysis of multimodal single-cell data. *Cell*, 184(13):3573–3587, 2021.

J. S. Herman, n. Sagar, and D. Gruen. FateID infers cell fate bias in multipotent progenitors from single-cell RNA-seq data. *Nature methods*, 15(5):379–386, 2018.

L. Heumos, A. C. Schaar, C. Lance, A. Litinetskaya, F. Drost, L. Zappia, M. D. Lücken, D. C. Strobl, J. Henao, F. Curion, et al. Best practices for single-cell analysis across modalities. *Nature Reviews Genetics*, pages 1–23, 2023.

G. Huguet, D. S. Magruder, A. Tong, O. Fasina, M. Kuchroo, G. Wolf, and S. Krishnaswamy. Manifold interpolating optimal-transport flows for trajectory inference. *Advances in Neural Information Processing Systems*, 35:29705–29718, 2022.

M. Ipsen. Evolutionary reconstruction of networks. *Function and regulation of cellular systems*, pages 241–249, 2004.

D. Kobak and P. Berens. The art of using t-SNE for single-cell transcriptomics. *Nature communications*, 10(1):5416, 2019.

G. La Manno, R. Soldatov, A. Zeisel, E. Braun, H. Hochgerner, V. Petukhov, K. Lidschreiber, M. E. Kastriti, P. Lönnerberg, A. Furlan, et al. RNA velocity of single cells. *Nature*, 560(7719):494–498, 2018.

W. V. Li and J. J. Li. A statistical simulator scDesign for rational scrna-seq experimental design. *Bioinformatics*, 35(14):i41–i50, 2019.

M. Marouf, P. Machart, V. Bansal, C. Kilian, D. S. Magruder, C. F. Krebs, and S. Bonn. Realistic in silico generation and augmentation of single-cell rna-seq data using generative adversarial networks. *Nature communications*, 11(1):166, 2020.

H. Matsumoto, H. Kiryu, C. Furusawa, M. S. Ko, S. B. Ko, N. Gouda, T. Hayashi, and I. Nikaido. SCODE: an efficient regulatory network inference algorithm from single-cell RNA-seq during differentiation. *Bioinformatics*, 33(15):2314–2321, 2017.

S. McCabe, L. Torres, T. LaRock, S. A. Haque, C.-H. Yang, H. Hartle, and B. Klein. netrd: A library for network reconstruction and graph distances. *arXiv preprint arXiv:2010.16019*, 2020.

A. Paszke, S. Gross, F. Massa, A. Lerer, J. Bradbury, G. Chanan, T. Killeen, Z. Lin, N. Gimelshein, L. Antiga, et al. Pytorch: An imperative style, high-performance deep learning library. *Advances in neural information processing systems*, 32, 2019.

X. Qiu, S. Ding, and T. Shi. From understanding the development landscape of the canonical fate-switch pair to constructing a dynamic landscape for two-step neural differentiation. *PloS one*, 7(12):e49271, 2012.

X. Qiu, Q. Mao, Y. Tang, L. Wang, R. Chawla, H. A. Pliner, and C. Trapnell. Reversed graph embedding resolves complex single-cell trajectories. *Nature methods*, 14(10):979–982, 2017.

X. Qiu, Y. Zhang, J. D. Martin-Rufino, C. Weng, S. Hosseinzadeh, D. Yang, A. N. Pogson, M. Y. Hein, K. H. J. Min, L. Wang, et al. Mapping transcriptomic vector fields of single cells. *Cell*, 185(4):690–711, 2022.

D. Risso, F. Perraudeau, S. Gribkova, S. Dudoit, and J.-P. Vert. A general and flexible method for signal extraction from single-cell RNA-seq data. *Nature communications*, 9(1):284, 2018.

W. Saelens, R. Cannoodt, H. Todorov, and Y. Saeys. A comparison of single-cell trajectory inference methods. *Nature biotechnology*, 37(5):547–554, 2019.

G. Schiebinger, J. Shu, M. Tabaka, B. Cleary, V. Subramanian, A. Solomon, J. Gould, S. Liu, S. Lin, P. Berube, et al. Optimal-transport analysis of single-cell gene expression identifies developmental trajectories in reprogramming. *Cell*, 176(4):928–943, 2019.

Y. Sha, Y. Qiu, P. Zhou, and Q. Nie. Reconstructing growth and dynamic trajectories from single-cell transcriptomics data. *Nature Machine Intelligence*, 6(1):25–39, 2024.

T. Sun, D. Song, W. V. Li, and J. J. Li. scDesign2: a transparent simulator that generates high-fidelity single-cell gene expression count data with gene correlations captured. *Genome biology*, 22(1):163, 2021.

A. Tong, J. Huang, G. Wolf, D. Van Dijk, and S. Krishnaswamy. Trajectorynet: A dynamic optimal transport network for modeling cellular dynamics. In *International conference on machine learning*, pages 9526–9536. PMLR, 2020.

F. W. Townes, S. C. Hicks, M. J. Aryee, and R. A. Irizarry. Feature selection and dimension reduction for single-cell RNA-seq based on a multinomial model. *Genome biology*, 20:1–16, 2019.

D. Tran, H. Nguyen, B. Tran, C. La Vecchia, H. N. Luu, and T. Nguyen. Fast and precise single-cell data analysis using a hierarchical autoencoder. *Nature communications*, 12(1):1029, 2021.

C. Trapnell, D. Cacchiarelli, J. Grimsby, P. Pokharel, S. Li, M. Morse, N. J. Lennon, K. J. Livak, T. S. Mikkelsen, and J. L. Rinn. The dynamics and regulators of cell fate decisions are revealed by pseudotemporal ordering of single cells. *Nature biotechnology*, 32(4):381–386, 2014.

P. Wang, C. Song, H. Zhang, Z. Wu, X.-J. Tian, and J. Xing. Epigenetic state network approach for describing cell phenotypic transitions. *Interface focus*, 4(3):20130068, 2014.

A. Woicik, M. Zhang, J. Chan, J. Ma, and S. Wang. Extrapolating heterogeneous time-series gene expression data using Sagittarius. *Nature Machine Intelligence*, pages 1–15, 2023.

F. A. Wolf, P. Angerer, and F. J. Theis. SCANPY: large-scale single-cell gene expression data analysis. *Genome biology*, 19:1–5, 2018.

F. A. Wolf, F. K. Hamey, M. Plass, J. Solana, J. S. Dahlin, B. Göttgens, N. Rajewsky, L. Simon, and F. J. Theis. PAGA: graph abstraction reconciles clustering with trajectory inference through a topology preserving map of single cells. *Genome biology*, 20:1–9, 2019.

R. Xiang, W. Wang, L. Yang, S. Wang, C. Xu, and X. Chen. A comparison for dimensionality reduction methods of single-cell RNA-seq data. *Frontiers in genetics*, 12:646936, 2021.

G. H. T. Yeo, S. D. Saksena, and D. K. Gifford. Generative modeling of single-cell time series with PRESCIENT enables prediction of cell trajectories with interventions. *Nature communications*, 12(1): 3222, 2021.

L. Zappia, B. Phipson, and A. Oshlack. Splatter: simulation of single-cell RNA sequencing data. *Genome biology*, 18(1):174, 2017.
